# Galilean Bulk-Surface Electrothermodynamics and Applications to Electrochemistry

**DOI:** 10.3390/e25030416

**Published:** 2023-02-25

**Authors:** Rüdiger Müller, Manuel Landstorfer

**Affiliations:** Weierstrass-Institute, Mohrenstr. 39, 10117 Berlin, Germany

**Keywords:** electrothermodynamics, bulk-surface systems, asymptotic analysis, entropy principle, constitutive modelling

## Abstract

In this work, the balance equations of non-equilibrium thermodynamics are coupled to Galilean limit systems of the Maxwell equations, i.e., either to (i) the quasi-electrostatic limit or (ii) the quasi-magnetostatic limit. We explicitly consider a volume Ω, which is divided into $\Omega^{+}$ and $\Omega^{-}$ by a possibly moving singular surface *S*, where a charged reacting mixture of a viscous medium can be present on each geometrical entity $\left(\Omega^{+},S,\Omega^{-}\right)$. By the restriction to the Galilean limits of the Maxwell equations, we achieve that only subsystems of equations for matter and electromagnetic fields are coupled that share identical transformation properties with respect to observer transformations. Moreover, the application of an entropy principle becomes more straightforward and finally helps estimate the limitations of the more general approach based the full set of Maxwell equations. Constitutive relations are provided based on an entropy principle, and particular care is taken in the analysis of the stress tensor and the momentum balance in the general case of non-constant scalar susceptibility. Finally, we summarise the application of the derived model framework to an electrochemical system with surface reactions.

## 1. Introduction

The reliable simulation of electrochemical systems on the device level requires thermodynamically consistent continuum models. Standard models such as the Poisson–Nernst–Planck system suffer from well-known limitations, cf., e.g., [1,2], of which the most-obvious deficiency is the missing volume exclusion effects. Several similar remedies for this problem have been proposed; see, e.g., [3,4,5] and the literature cited therein. More recent approaches that also take the dielectric effects of the solvent into account were given, e.g., in [6,7]. The latter one builds on a bulk-surface electrothermodynamic modelling framework summarised in [8], which unifies classical non-equilibrium thermodynamics containing electromagnetic fields as in [9,10] and its extensions to surfaces [11,12]. As an alternative modelling framework, Reference [13] recently developed a multiscale electrothermodynamics approach within the GENERIC formalism. Although not less complex and technical compared to [8], this approach is so far limited to closed bulk systems.

Applying the abstract framework of [8] to electrochemical problems typically first requires strong simplifications of the model, thereby favouring the impression that the framework appears too general and not precisely tailored to the considered class of problems. Moreover, the readers of [8] might be confused by some decisions made there for mainly pragmatic reasons, but leading to a discussion of the stability conditions that are related to the choice of independent variables and that are necessary in addition to the employed entropy principle. Finally, the way that the electromagnetic sub-system and the classical material mixture part are coupled in [8] might not appear fully satisfactory because of the different transformation properties of these sub-systems. The problem of different transformation properties can be remedied in two alternative ways. On the one hand, one can give up mass conservation in favour of a fully relativistic description of matter. On the other hand, one may try to couple a low-velocity Galilean limit system of the Maxwell equations to the classical, i.e., non-relativistic, balance equations of mixtures of matter.

In this work, we proceed with the second of the alternatives, since we aim at engineering applications and, in particular, electrochemical applications. As shown by [14], cf. also [15], there is not one unique Galilean limit of the Maxwell equations, but instead, there are two distinct limits, i.e., the electric—or quasi-electrostatic—limit and the magnetic—or quasi-magnetostatic—limit. While coupling the electric limit to the balance equations of mass, momentum, and energy seems most reasonable for electrochemical applications, the magnetic Galilean limit is more naturally related to magneto-hydrodynamics. We re-examine the derivation of Galilean limit systems of the Maxwell equations here, as we are not aware that this has been carried out before for the case of singular surfaces. This way, we can make sure that we do not miss relevant effects during coupling to the balances of matter later on or during the following derivation of constitutive equations. For each of the two Galilean limit cases considered in this work, there is one corresponding variant of the entropy principle in [8] such that coupling the general full set of Maxwell equations to the classical balance equations of matter and taking the Galilean limit after exploitation of the entropy principle yield the same results as obtained here. However, none of the two alternative variants of the entropy principle in [8] are capable of covering both Galilean limit cases equally well. It turns out that the application of the entropy principle here is much more straightforward compared to [8], as a re-definition of the combined inner energy of field and matter becomes unnecessary and a discussion of the stability of the polarisation relaxation does not arise.

The obtained constitutive equations differ between the two Galilean limit cases. As can be expected, polarisation relaxation is only covered in the quasi-electrostatic case, whereas the relaxation of magnetisation is only present in the quasi-magnetostatic case. Moreover, the generalised driving forces for thermodiffusion differ between the Galilean limits, as does the stress tensor.

We apply the geometrical setting and notation of [8], but neglected the elastic deformations, since these effects do not contribute to the questions discussed here. The results obtained here can be generalised in a safe and straightforward manner to include elastic effects in the same way as in [8].

## 2. Notation

We consider a geometrical setup where, locally, an orientable surface *S* divides a domain $\Omega \subset R^{3}$ into two subdomains $\Omega^{\pm }\subseteq R^{3}$ with $S:=\partial \Omega^{+}\cap \partial \Omega^{-}$. The domain Ω, as well as the surface *S* may evolve in time. In addition to quantities defined in the domains $\Omega^{+}$ or $\Omega^{-}$, there are, in general, corresponding quantities on the surfaces *S*, which, in general, do not coincide with the corresponding traces from the subdomains. As a convention, the same letters are used for these quantities, but the surface variables are indicated by an underset *s*.

We apply the convention of implicit summation over coordinate indices appearing twice. We indicate the Cartesian components of vectors and tensors by lowercase Latin indices, e.g., i,j, whereas at the surface *S*, we use uppercase Greek indices such as, e.g., Γ,Δ for the tangential components.

The tangential vectors are defined as the partial derivatives of the smooth bijective parametrisation θ mapping from some open parameter domain to the surface *S*. In addition, we define the (unit) normal vector and the metric tensor by

(1) $$\tau_{1/2}=\frac{\partial \theta (t,U^{1},U^{2})}{\partial U^{1/2}}\;,\;\nu =\frac{\tau_{1}\times \tau_{2}}{|\tau_{1}\times \tau_{2}|}\;\;\mathrm{and}\;\;g={\left[\tau_{1},\tau_{2}\right]}^{T}\left[\tau_{1},\tau_{2}\right]\;.$$

As a convention, we chose the mapping θ such that ν is the inner normal of $\Omega^{+}$. For the matrix components of the metric tensor g, we use lower indices $g_{\Delta \Gamma }$, and we use upper indices $g^{\Delta \Gamma }$ for the components of the inverse matrix of the metric. A vector V defined on the surface can, thus, be written as $V=V_{\tau }^{\Delta }\tau_{\Delta }+V_{\nu }\nu$, with the normal component by $V_{\nu }$ and the tangential components $V_{\tau }^{\Delta }=g^{\Gamma \Delta }V\tau_{\Gamma }$, for Δ=1,2.

The *curvature tensor* $b_{\Delta \Gamma }$ of the surface *S* and the *Christoffel symbols* $\Gamma_{\Delta \Gamma }^{\Sigma }$ are defined by a decomposition of the derivatives of the tangential vectors into their tangential and normal components:

(2) $$\frac{\partial \tau_{\Delta }}{\partial U^{\Gamma }}=\Gamma_{\Delta \Gamma }^{\Sigma }\;\tau_{\Sigma }+b_{\Delta \Gamma }\;\nu \;\;\mathrm{for}\;\Gamma ,\Delta =1,2\;.$$

Then, the mean curvature of *S* is $k_{M}=\frac{1}{2}b_{\Gamma \Delta }g^{\Gamma \Delta }$. Let a:S→R be a scalar and $V:S\to R^{3}$ a vector field. Then, the covariant derivatives of the tangential components are defined as

(3) $$\begin{array}{ccccc} a_{∥\Gamma } & =\frac{\partial a}{\partial U^{\Gamma }}\;,\;\;\mathrm{for}\;\Gamma =1,2\;, & V_{\tau ∥\Gamma }^{\Delta } & =\frac{\partial V_{\tau }^{\Delta }}{\partial U^{\Gamma }}+\Gamma_{\Gamma \Sigma }^{\Delta }\;V_{\tau }^{\Sigma }\;\;\mathrm{for}\;\Gamma ,\Delta =1,2\;. & \end{array}$$

Let $\underset{s}{\upsilon }\;\;$ denote the velocity of the surface *S*. For a scalar $a:[0,t_{\mathrm{end}})\times S\to R$, we define the time derivative:

(4) $$\partial_{t,\nu }a=\partial_{t}a-a_{∥\Delta }\;\underset{s}{\upsilon }\;{\;}_{\tau }^{\Delta }\;.$$

Let *u* be a generic function defined at least in one of the subdomains $\Omega^{\pm }$. We denote the *trace* of *u* by

(5) $$u^{\pm }\left(t,\underset{s}{x}\;\;\right)=\lim_{x\in \Omega^{\pm }\to \underset{s}{x}\;\;\in S}u\left(t,x\right)\;,$$

whenever *u* is defined on this side of the surface; otherwise, we set the corresponding trace to zero. We define the *jump* and the *mean value* of *u* at the surface *S* by

(6) $$\begin{array}{cccc} \left[\;[u]\;\right] & =u^{+}-u^{-}\;, & \bar{u} & =\frac{1}{2}\left(u^{+}+u^{-}\right). \end{array}$$

## 3. Maxwell Equations and Their Galilean Limits

The Maxwell equations for the electromagnetic field are most generally formulated such that time and space and all involved equations are properly combined into four-dimensional objects. Then, the equations satisfy the most-fundamental symmetry principle, i.e., the principle of relativity, meaning that the balance equations, as well as the constitutive equations remain invariant with respect to arbitrary observer transformations.

Here, 1+3-dimensional Maxwell equations are formulated in a way following the classical work of Truesdell and Toupin [16]. This includes the postulation of Maxwell–Lorentz aether relations that are independent of the considered material. The precise form of these relations depends on the frame of reference. Here and in the following section, we assume an inertial frame of reference such that the Maxwell–Lorentz aether relations take the most-simple form (9) below. In the 1+3-dimensional setting, Maxwell–Lorentz aether relations are only invariant with respect to Lorentz transformations. The Galilean transformation is a good approximation of the Lorentz transformation in the limit of vanishing barycentric velocity, i.e., $v/c_{0}\to 0$. However, the derivation of a Galilean limit system of the Maxwell equations is complicated by the fact that there are two different limit systems, cf. [14,15].

### 3.1. General Maxwell Equations

The underlying physical principle for the derivation of the Maxwell equations is the conservation of charge and magnetic flux. The *(total) electric charge* density $n^{e}$ in each volume domain $\Omega^{\pm }$, a well as $\underset{s}{n}\;{\;}^{e}$ on the surface satisfy the local conservation equations, viz.

(7a) $$\begin{array}{ccc} \partial_{t}n^{e}+\mathrm{div}\left(n^{e}\upsilon +J^{e}\right) & =0\;, & \end{array}$$

(7b) $$\begin{array}{cc} \partial_{t,\nu }\underset{s}{n}\;{\;}^{e}+{\left(\underset{s}{n}\;{\;}^{e}\underset{s}{\upsilon }\;{\;}_{\tau }^{\Delta }+\underset{s}{J}\;{\;}_{\tau }^{e,\Delta }\right)}_{∥\Delta }-2k_{M}\underset{s}{\upsilon }\;{\;}_{\nu }\;\underset{s}{n}\;{\;}^{e}\; & =-[\;\left[n^{e}\left(\upsilon_{\nu }-\underset{s}{\upsilon }\;{\;}_{\nu }\right)+J_{\nu }^{e}\right]\;]\;. \end{array}$$

Here, we split the *electric current* as $j^{e}=n^{e}\upsilon +J^{e}$. We introduce the *charge potential* D and the *current potential* H by means of a formal solution of the charge balances (7); see [8] Therefore, we have

(8a) $$\begin{array}{cccc} n^{e} & =\mathrm{div}(D)\;, & \;J^{e} & =-\partial_{t}D-\upsilon \mathrm{div}\left(D\right)+\mathrm{curl}\left(H\right)\;, \end{array}$$

(8b) $$\begin{array}{cccc} \underset{s}{n}\;{\;}^{e} & =[\;[D\cdot \nu ]\;]\;, & \;\underset{s}{J}\;{\;}^{e} & =\nu \times [\;\left[H-\underset{s}{\upsilon }\;\;\times D\right]\;]\;. \end{array}$$

The conservation of *magnetic flux* in the bulk and on the surface reads

(8c) $$\begin{array}{cccc} 0 & =\mathrm{div}(B)\;, & 0 & =\partial_{t}B+\mathrm{curl}\left(E\right)\;, \end{array}$$

(8d) $$\begin{array}{cccc} 0 & =[\;[B\cdot \nu ]\;]\;, & 0 & =\nu \times [\;\left[E+\underset{s}{\upsilon }\;\;\times B\right]\;]\;, \end{array}$$

where E is the electric field. We postulate universal valid Maxwell–Lorentz aether relations, i.e., independent of the considered material, it holds in an inertial frame:

(9) $$\begin{array}{c} D=\varepsilon_{0}E\;\mathrm{and}\;H=\frac{1}{\mu_{0}}B\;, \end{array}$$

where the *dielectric constant* $\varepsilon_{0}$ and the *magnetic constant* $\mu_{0}$ are related to the speed of light by $\varepsilon_{0}\mu_{0}=c_{0}^{-2}$. The remaining constitutive quantity is the electric flux density $J^{e}$.

As an immediate consequence of the universal valid Maxwell–Lorentz aether relations (9) and charge conservation (7a), we conclude that the sum of the electric current and the displacement current is at any time *t* and location x source-free. This motivates the definition of the total current as

(10) $$\begin{array}{cccc} j & :=j^{e}+\varepsilon_{0}\partial_{t}E\; & ⟹\;\mathrm{div}(j) & =0\;. \end{array}$$

#### Implied Balances

From Maxwell’s Equation (8), the following two additional balance equations can be derived, cf. [10]. The balance of electromagnetic momentum reads in each subdomain $\Omega^{\pm }$, as well as on the surface *S*

(11a) $$\begin{array}{ccccc} \partial_{t}\left(D\times B\right)-\mathrm{div}\left(\sigma^{\mathrm{EM}}\right)\; & =-n^{e}E-\left(n^{e}\upsilon +J^{e}\right)\times B & \; & =:-k\;, & \end{array}$$

(11b) $$\begin{array}{cccc} -[\;\left[D\times B\right)\underset{s}{\upsilon }\;{\;}_{\nu }]\;]+\left[\;[\sigma^{\mathrm{EM}}\;\nu ]\;\right]\; & =-\underset{s}{n}\;{\;}^{e}\bar{E}-\left(\underset{s}{n}\;{\;}^{e}\underset{s}{\upsilon }\;\;+\underset{s}{J}\;{\;}^{e}\right)\times \bar{B} & \; & =:-\underset{s}{k}\;\;\;, \end{array}$$

where the right-hand sides are the negative Lorentz force in the volume and on the surface, respectively. The *Maxwell* stress tensor in (11) is

(12) $$\begin{array}{cc} \sigma^{\mathrm{EM}}\; & =E⊗D-\frac{1}{2}E\cdot D\;1\;+H⊗B-\frac{1}{2}H\cdot B\;1\;, \end{array}$$

where 1 denotes the identity matrix. The balance of electromagnetic energy reads

(13a) $$\begin{array}{ccccc} \frac{1}{2}\partial_{t}\left(E\cdot D+B\cdot H\right)+\mathrm{div}\left(E\times H\right)\; & =-(n^{e}\upsilon +J^{e})\cdot E & \; & =:-\pi \;, & \end{array}$$

(13b) $$\begin{array}{cccc} -\frac{1}{2}\left[\;\left[\left(E\cdot D+B\cdot H\right)\underset{s}{\upsilon }\;{\;}_{\nu }\right]\;\right]+\left[\;\left[\left(E\times H\right)\cdot \nu \right]\;\right] & =-\left(\underset{s}{n}\;{\;}^{e}\underset{s}{\upsilon }\;\;+\underset{s}{J}\;{\;}^{e}\right)\cdot \bar{E} & \; & =:-\underset{s}{\pi }\;\;\;, \end{array}$$

where the right-hand sides are the negative Joule heat in the volume and on the surface, respectively.

### 3.2. Polarisation and Magnetisation

When considering a macroscopic non-relativistic continuum description of mixtures of charged matter, there is the *free charge* density $n^{F}$ defined as a Galilean conserved quantity. In general, this free charge does not coincide with the previously introduced electric charge $n^{e}$. As an example, $n^{F}$ may solely represent the net charge of molecules in a mixture without taking into account the internal electronic structure. However, also this internal electronic structure in general has relevance for the overall electromagnetic field. To bridge this gap and represent these microscopic effects on the more macroscopic level, we introduce the *polarisation charge density* $n^{P}$ and the *polarisation current density* $J^{P}$ as

(14) $$\begin{array}{cccc} n^{e} & =n^{F}+n^{P}\;, & J^{e} & =J^{F}+J^{P}\;, \end{array}$$

The conservation of the electric charge $n^{e}$ and of the free charge $n^{F}$ then also imply the conservation of the polarisation charge $n^{P}=n^{e}-n^{F}$. We introduce the vector of *polarisation* P and *Lorentz magnetisation* M by the same approach of the formal solution to the conservation equations, viz.

(15a) $$\begin{array}{ccccc} n^{P} & =-\mathrm{div}(P), & \; & J^{P} & =\partial_{t}P+\upsilon \mathrm{div}\left(P\right)+\mathrm{curl}\left(M\right)\;, \end{array}$$

(15b) $$\begin{array}{ccccc} \underset{s}{n}\;{\;}^{P} & =-[\;[P]\;]\cdot \nu , & \; & \underset{s}{J}\;{\;}^{P} & =\nu \times [\;\left[M+\underset{s}{\upsilon }\;\;\times P\right]\;]\;. \end{array}$$

Not all of the introduced quantities so far are Galilean scalars, vectors, or tensors, respectively. Due to its importance in electrodynamics and for the upcoming constitutive modelling, we introduce the Galilean vectors of the *electromotive intensity* and the *magnetisation*:

(16) $$\begin{array}{ccccc} E & =E+\upsilon \times B\;, & \; & M & =M+\upsilon \times P\;. \end{array}$$

The electric current can then be expressed as

(17) $$\begin{array}{cc} j^{e} & =n^{F}\upsilon +J^{F}+\partial_{t}P+\mathrm{curl}\left(M-\upsilon \times P\right)\;. \end{array}$$

For later use in the balance of the inner energy, we verify that

(18a) $$\begin{array}{cc} \pi -k\cdot \upsilon \; & =(J^{F}+J^{P})\cdot E \end{array}$$

(18b) $$\begin{array}{cc} \underset{s}{\pi }\;\;-\underset{s}{k}\;\;\cdot \underset{s}{\upsilon }\;\;\; & =\left(\underset{s}{J}\;{\;}^{F}+\underset{s}{J}\;{\;}^{P}\right)\cdot \left(\bar{E}+\underset{s}{\upsilon }\;\;\times \bar{B}\right)\;. \end{array}$$

### 3.3. Non-Dimensional System and Quasi-Static Galilean Limits

#### Non-Dimensional Form of the System

To write the model equations in non-dimensional form, we introduce characteristic reference values $t^{\mathrm{ref}},x^{\mathrm{ref}}$ for the time and space coordinates. Velocity is then scaled by the derived reference value $\upsilon^{\mathrm{ref}}=x^{\mathrm{ref}}/t^{\mathrm{ref}}$, cf. Table 1. However, we remark that a different scaling of velocity might be more appropriate in a system coupled to the diffusion of matter. We introduce reference values $E^{\mathrm{ref}},B^{\mathrm{ref}}$ for the electric and magnetic field strength, respectively. Moreover, we use the elementary charge $e_{0}$ as the reference value for charge and introduce $T^{\mathrm{ref}}$ and $n_{\alpha }^{\mathrm{ref}}$ as the reference values for the temperature and for the number densities of particles in the volume domains, respectively.

Upon the definition of the dimensionless constants:

(19) $$\begin{array}{cccccccc} \beta & =\sqrt{\frac{c_{0}B^{\mathrm{ref}}}{E^{\mathrm{ref}}}}\;, & \; & \; & \lambda & =\sqrt{\frac{\varepsilon_{0}E^{\mathrm{ref}}}{e_{0}n^{\mathrm{ref}}x^{\mathrm{ref}}}}\;, & \kappa & =\sqrt{\frac{e_{0}\;c_{0}B^{\mathrm{ref}}\;x^{\mathrm{ref}}}{k_{B}T^{\mathrm{ref}}}}\;, \end{array}$$

we obtain in the volume domains $\Omega^{\pm }$ the following dimensionless system:

(20a) $$\begin{array}{cc} \lambda^{2}\mathrm{div}\left(\overset{˘}{E}+\overset{˘}{P}\right) & ={\overset{˘}{n}}^{F}\;, \end{array}$$

(20b) $$\begin{array}{cc} -\lambda^{2}\partial_{t}\left(\overset{˘}{E}+\overset{˘}{P}\right)+\lambda^{2}\frac{c_{0}}{\upsilon^{\mathrm{ref}}}\beta^{2}\mathrm{curl}\left(\overset{˘}{B}-\overset{˘}{M}\right) & ={\overset{˘}{n}}^{F}\overset{˘}{\upsilon }+{\overset{˘}{J}}^{F}\;, \end{array}$$

(20c) $$\begin{array}{cc} \mathrm{div}(\overset{˘}{B}) & =0\;, \end{array}$$

(20d) $$\begin{array}{cc} \beta^{2}\frac{\upsilon^{\mathrm{ref}}}{c_{0}}\partial_{t}\overset{˘}{B}+\mathrm{curl}\left(\overset{˘}{E}\right) & =0\;. \end{array}$$

For the surface, we introduce an additional reference number density $\underset{s}{n}\;{\;}^{\mathrm{ref}}$ and relate it to the volume by the dimensionless constant:

(21) $$\begin{array}{cc} \delta & =\frac{\underset{s}{n}\;{\;}^{\mathrm{ref}}}{n^{\mathrm{ref}}\;\lambda x^{\mathrm{ref}}}\;. \end{array}$$

Then, the surface balances are in dimensionless form:

(22a) $$\begin{array}{ccc} \lambda [\;\left[\left(\overset{˘}{E}+\overset{˘}{P}\right)\cdot \nu \right]\;] & =\delta \overset{˘}{\underset{s}{n}\;\;}{}^{F}\;, & \end{array}$$

(22b) $$\begin{array}{cc} \lambda \nu \times [\;\left[\frac{c_{0}}{\upsilon^{\mathrm{ref}}}\beta^{2}\left(\overset{˘}{B}-\overset{˘}{M}\right)-\overset{˘}{\underset{s}{\upsilon }\;\;}\times \left(\overset{˘}{E}+\overset{˘}{P}\right)\right]\;] & =\delta \overset{˘}{\underset{s}{J}\;\;}{}^{F}\;, \end{array}$$

(22c) $$\begin{array}{ccc} \left[\;\left[\overset{˘}{B}\cdot \nu \right]\;\right] & =0\;, & \end{array}$$

(22d) $$\begin{array}{cc} \nu \times [\;\left[\overset{˘}{E}+\beta^{2}\frac{\upsilon^{\mathrm{ref}}}{c_{0}}\overset{˘}{\underset{s}{\upsilon }\;\;}\times \overset{˘}{B}\right]\;] & =0\;. \end{array}$$

The balance of electromagnetic momentum reads in the volume domains and on the surface

(23a) $$\begin{array}{cc} \beta^{2}\partial_{t}\left(\kappa^{2}\lambda^{2}\frac{\upsilon^{\mathrm{ref}}}{c_{0}}\overset{˘}{E}\times \overset{˘}{B}\right)+\mathrm{div}\left(-\beta^{2}\overset{˘}{\sigma }{}^{\mathrm{EM}}\right)\; & =-\beta^{2}\overset{˘}{k}\;, \end{array}$$

(23b) $$\begin{array}{cc} -\beta^{2}\left[\;\left[\kappa^{2}\lambda^{2}\frac{\upsilon^{\mathrm{ref}}}{c_{0}}\cdot \left(\overset{˘}{E}\times \overset{˘}{B}\right)\underset{s}{\overset{˘}{\upsilon }}\;{\;}_{\nu }\right]\;\right]+\left[\;\left[\beta^{2}\overset{˘}{\sigma }{}^{\mathrm{EM}}\;\nu \right]\;\right]\; & =-\lambda \delta \beta^{2}\overset{˘}{\underset{s}{k}\;\;}\;. \end{array}$$

Here, the Maxwell stress tensor multiplied by $\beta^{2}$, viz.

(24) $$\begin{array}{cc} \; & \beta^{2}{\overset{˘}{\sigma }}^{\mathrm{EM}}=\kappa^{2}\lambda^{2}\cdot \left(\overset{˘}{E}⊗\overset{˘}{E}-\frac{1}{2}\overset{˘}{E}\cdot \overset{˘}{E}1+\beta^{4}\left(\overset{˘}{B}⊗\overset{˘}{B}-\frac{1}{2}\overset{˘}{B}\cdot \overset{˘}{B}1\right)\right) \end{array}$$

contains terms independent of $\beta^{2}$, as well as the Lorentz force and the Joule heat do, i.e.,

(25a) $$\begin{array}{ccccc} \beta^{2}\overset{˘}{k} & =\kappa^{2}\cdot \left({\overset{˘}{n}}^{e}\overset{˘}{E}+\beta^{2}\frac{\upsilon^{\mathrm{ref}}}{c_{0}}{\overset{˘}{J}}^{e}\times \overset{˘}{B}\right)\;, & \beta^{2}\overset{˘}{\pi } & =\kappa^{2}\cdot \left({\overset{˘}{n}}^{e}\overset{˘}{E}\cdot \overset{˘}{\upsilon }+{\overset{˘}{J}}^{e}\cdot \overset{˘}{E}\right)\;, & \end{array}$$

(25b) $$\begin{array}{cccc} \beta^{2}\underset{s}{\overset{˘}{k}}\;\; & =\kappa^{2}\cdot \left(\underset{s}{\overset{˘}{n}}\;\;{}^{e}\bar{\overset{˘}{E}}+\beta^{2}\frac{\upsilon^{\mathrm{ref}}}{c_{0}}\left(\underset{s}{\overset{˘}{n}}\;\;{}^{e}\underset{s}{\overset{˘}{\upsilon }}\;\;+\underset{s}{\overset{˘}{J}}\;{\;}^{e}\right)\times \bar{\overset{˘}{B}}\right)\;, & \beta^{2}\underset{s}{\overset{˘}{\pi }}\;\; & =\kappa^{2}\cdot \left(\underset{s}{\overset{˘}{n}}\;\;{}^{e}\bar{\overset{˘}{E}}\cdot \underset{s}{\overset{˘}{\upsilon }}\;\;+\underset{s}{\overset{˘}{J}}\;\;{}^{e}\cdot \bar{\overset{˘}{E}}\right)\;. \end{array}$$

The electromotive intensity and magnetisation are

(26) $$\begin{array}{ccccc} \overset{˘}{E} & =\overset{˘}{E}+\beta^{2}\frac{\upsilon^{\mathrm{ref}}}{c_{0}}\left(\overset{˘}{\upsilon }\times \overset{˘}{B}\right)\;, & \; & \overset{˘}{M} & =\overset{˘}{M}+\frac{\upsilon^{\mathrm{ref}}}{c_{0}}\frac{1}{\beta^{2}}\left(\overset{˘}{\upsilon }\times \overset{˘}{P}\right)\;, \end{array}$$

The balance of electromagnetic energy reads

(27a) $$\begin{array}{ccc} \frac{1}{2}\lambda^{2}\kappa^{2}\partial_{t}\left(\frac{1}{\beta^{2}}|\overset{˘}{E}{|}^{2}+\beta^{2}{\left|\overset{˘}{B}\right|}^{2}\right)+\lambda^{2}\kappa^{2}\mathrm{div}\left(\overset{˘}{E}\times \overset{˘}{B}\right)\; & =-\overset{˘}{\pi }\;, & \end{array}$$

(27b) $$\begin{array}{cc} -\frac{1}{2}\lambda \kappa^{2}[\;[(\frac{1}{\beta^{2}}|\overset{˘}{E}{|}^{2}+\beta^{2}|\overset{˘}{B}{|}^{2})\underset{s}{\upsilon }\;{\;}_{\nu }]\;]+\lambda \kappa^{2}\left[\;\left[\left(\overset{˘}{E}\times \overset{˘}{B}\right)\cdot \nu \right]\;\right]\; & =-\delta \;\underset{s}{\overset{˘}{\pi }}\;\;\;, \end{array}$$

and the introduction of polarisation and Lorentz magnetisation implies, for the electric current and the surface electric charge flux,

(28a) $$\begin{array}{ccc} \overset{˘}{j}{}^{e} & ={\overset{˘}{n}}^{F}\overset{˘}{\upsilon }+\overset{˘}{J}{}^{F}+\lambda^{2}\partial_{t}\overset{˘}{P}+\lambda^{2}\mathrm{curl}\left(\frac{c_{0}}{\upsilon^{\mathrm{ref}}}\beta^{2}\overset{˘}{M}-\overset{˘}{\upsilon }\times \overset{˘}{P}\right)\;, & \end{array}$$

(28b) $$\begin{array}{cc} \delta \overset{˘}{\underset{s}{J}\;\;}{}^{e} & =\lambda \nu \times [\;\left[\frac{c_{0}}{\upsilon^{\mathrm{ref}}}\beta^{2}\overset{˘}{B}-\overset{˘}{\underset{s}{\upsilon }\;\;}\times \overset{˘}{E}\right]\;]\;. \end{array}$$

Depending on the chosen characteristic reference values $t^{\mathrm{ref}}$, $x^{\mathrm{ref}}$, $E^{\mathrm{ref}},B^{\mathrm{ref}}$, $T^{\mathrm{ref}}$, $n_{\alpha }^{\mathrm{ref}}$, and $\underset{s}{n}\;{\;}_{\alpha }^{\mathrm{ref}}$, the size of the dimensionless quantities may differ by several orders of magnitude, allowing considerable simplifications of the model equations. We consider in the following two alternative limiting cases for the parameter β, whereas we only assume for the remaining parameters λ, κ, ω, and δ that they remain moderate in size. At this point, we only remark that, later on, it is possible to analyse additional limit processes, such as, e.g., λ→0, which corresponds to the thin interface limit in electrochemical applications.

### 3.4. Quasi-Electrostatic Limit $\beta^{2}\ll 1$

We assume that β is a small parameter, whereas $\frac{v^{\mathrm{ref}}}{c_{0}}$ and the time derivative of B remain bounded. As a consequence, we obtain the equations of electrostatics for the electric field, which read in the dimensional form

(29a) $$\begin{array}{cccccc} \mathrm{div}(\varepsilon_{0}E+P) & =n^{F}\;, & \; & \left[\;\left[\left(\varepsilon_{0}E+P\right)\cdot \nu \right]\;\right] & =\underset{s}{n}\;{\;}^{F}\;, & \end{array}$$

(29b) $$\begin{array}{ccccc} \mathrm{curl}(E) & =0\;, & \; & \nu \times [\;[E]\;] & =0\;. \end{array}$$

A constitutive equation for P, which is independent of B and M, then allows for a given free charge obtaining E and P from (29). The balance of electromagnetic momentum reduces to

(30) $$\begin{array}{ccccc} -\mathrm{div}\left(\sigma^{\mathrm{EM}}\right) & =-k\;, & \; & +[\;\left[\sigma^{\mathrm{EM}}\;\nu \right]\;] & =-\underset{s}{k}\;\;\;, \end{array}$$

with the Maxwell stress tensor depending only on E, viz.

(31) $$\begin{array}{cc} \sigma^{\mathrm{EM}} & =\varepsilon_{0}\left(E⊗E-\frac{1}{2}{\left|E\right|}^{2}\;1\right)\;. \end{array}$$

In this limit, the electromotive intensity is identical to the electric field. The magnetic field is not vanishing; it does not have any effect on the Lorentz force, viz.

(32a) $$\begin{array}{ccccccc} E & =E\;, & k & =n^{e}E\;, & \pi & =n^{e}E\cdot \upsilon +J^{e}\cdot E\;, & \end{array}$$

(32b) $$\begin{array}{cccccc} \; & \; & \underset{s}{k}\;\; & =\underset{s}{n}\;{\;}^{e}\bar{E}\;, & \underset{s}{\pi }\;\; & =\underset{s}{n}\;{\;}^{e}\bar{E}\cdot \underset{s}{\upsilon }\;\;+\underset{s}{J}\;{\;}^{e}\cdot \bar{E}\;. \end{array}$$

The Joule heat cannot be evaluated from E and P alone, but also requires knowledge of B. The remaining Maxwell equations, in general, together with a constitutive equation for M, determine the magnetic flux density B, i.e.,

(33a) $$\begin{array}{ccccc} \mathrm{div}(B) & =0\;, & \partial_{t}\left(\varepsilon_{0}E+P\right)+n^{F}\upsilon +J^{F} & =\mathrm{curl}(\frac{1}{\mu_{0}}B-M+\upsilon \times P)\;, & \end{array}$$

(33b) $$\begin{array}{cccc} \left[\;[B\cdot \nu ]\;\right] & =0\;, & \nu \times \left[\;\left[\underset{s}{\upsilon }\;\;\times \left(\varepsilon_{0}E+P\right)\right]\;\right]+\underset{s}{J}\;{\;}^{F} & =\nu \times [\;\left[\frac{1}{\mu_{0}}B-M+\upsilon \times P\right]\;]\;. \end{array}$$

The electric current and the surface current flux are then

(34) $$\begin{array}{cccc} j^{e} & =n^{F}\upsilon +J^{F}+\partial_{t}P+\mathrm{curl}\left(M-\upsilon \times P\right)\;, & \underset{s}{J}\;{\;}^{e} & =\nu \times [\;\left[\frac{1}{\mu_{0}}B-\underset{s}{\upsilon }\;\;\times \left(\varepsilon_{0}E\right)\right]\;]\;. \end{array}$$

### 3.5. Quasi-Magentostatic Limit $\frac{1}{\beta^{2}}\ll 1$

Under the assumption that the derivatives of $\overset{˘}{E}+\overset{˘}{P}$ remain bounded, Maxwell’s equations simplify in this limit to

(35a) $$\begin{array}{cccccc} \mathrm{curl}(\frac{1}{\mu_{0}}B-M) & =n^{F}\upsilon +J^{F}\;, & \; & \nu \times [\;\left[\frac{1}{\mu_{0}}B-M\right]\;] & =\underset{s}{J}\;{\;}^{F}\;, & \end{array}$$

(35b) $$\begin{array}{ccccc} \mathrm{div}(B) & =0\;, & \; & \left[\;[B\cdot \nu ]\;\right] & =0\;. \end{array}$$

A constitutive equation for M, which is independent of E and P, then allows for a given free current to obtain B and M from (35). The balance of electromagnetic momentum reduces to

(36) $$\begin{array}{ccccc} -\mathrm{div}\left(\sigma^{\mathrm{EM}}\right) & =-k\;, & \; & +[\;\left[\sigma^{\mathrm{EM}}\;\nu \right]\;] & =-\underset{s}{k}\;\;\;, \end{array}$$

with the Maxwell stress tensor depending only on B, viz.

(37) $$\begin{array}{cc} \sigma^{\mathrm{EM}} & =\frac{1}{\mu_{0}}\left(B⊗B-\frac{1}{2}{\left|B\right|}^{2}\;1\right)\;. \end{array}$$

In the quasi-magnetostatic limit, the magnetisation is identical to the Lorentz magnetisation. The electric field does not vanish; it does not have any effect on the Lorentz force, and the Joule heat is negligible, viz.

(38a) $$\begin{array}{cccccc} M & =M\;, & k & =(n^{e}\upsilon +J^{e})\times B\;, & \pi -k\cdot \upsilon & =J^{e}\cdot \left(\upsilon \times B\right)\;, \end{array}$$

(38b) $$\begin{array}{cccccc} \; & \; & \underset{s}{k}\;\; & =\left(\underset{s}{n}\;{\;}^{e}\underset{s}{\upsilon }\;\;+\underset{s}{J}\;{\;}^{e}\right)\times \bar{B}\;, & \underset{s}{\pi }\;\;-\underset{s}{k}\;\;\cdot \underset{s}{\upsilon }\;\; & =\underset{s}{J}\;{\;}^{e}\cdot \left(\underset{s}{\upsilon }\;\;\times \bar{B}\right)\;. \end{array}$$

The Lorentz force cannot be evaluated from B and M alone, but also requires knowledge of E. The remaining Maxwell equations, together with a constitutive equation for P, determine the electric field, i.e.,

(39a) $$\begin{array}{cccccc} \partial_{t}B+\mathrm{curl}\left(E\right) & =0\;, & \; & \nu \times [\;\left[E+\underset{s}{\upsilon }\;\;\times B\right]\;] & =0\;, & \end{array}$$

(39b) $$\begin{array}{ccccc} \mathrm{div}(\varepsilon_{0}E+P) & =n^{F}\;, & \; & \left[\;\left[\left(\varepsilon_{0}E+P\right)\cdot \nu \right]\;\right] & =\underset{s}{n}\;{\;}^{F}\;. \end{array}$$

The electric current and the surface current flux are

(40) $$\begin{array}{ccccc} j^{e} & =n^{F}\upsilon +J^{F}+\partial_{t}P+\mathrm{curl}\left(M\right)\;, & \; & \underset{s}{J}\;{\;}^{e} & =\nu \times [\;\left[\frac{1}{\mu_{0}}B\right]\;]\;. \end{array}$$

## 4. Balance Equations of Galilean Electrothermodynamics

The Galilean limit systems of electromagnetics can now be consistently coupled to classical, i.e., non-relativistic balance equations for charged mixtures of matter. We consider partial mass balances for each of the constituents of the mixture, a single momentum balance of the mixture, and an energy balance of matter.

### 4.1. Description of Reacting Mixtures

We use different index sets $I^{\pm }$ to refer to the constituents of a mixture in $\Omega^{\pm }$ and the index set $I_{S}$ for the constituents on the surface *S*. We apply the non-restrictive assumption that the sets $I^{\pm }$ are disjoint, i.e., $I^{+}\cap I^{-}=\emptyset$. All constituents of the subdomains $\Omega^{\pm }$ are assumed to be also constituents on the surface *S*, but there may be some additional constituents that are exclusively present on *S*. Thus, we have $I^{\pm }\subseteq I_{S}$.

There may be several chemical reactions among the bulk constituents, as well as chemical reactions on the surface. Picking some indices k,ℓ, then, these reactions in the bulk or surface may be written in the general form:

(41) $$\begin{array}{ccccc} \sum_{\alpha \in I^{\pm }}a_{\alpha }^{k}{\begin{array}{c} A \end{array}}_{\alpha } & ⇌\sum_{\alpha \in I^{\pm }}b_{\alpha }^{k}{\begin{array}{c} A \end{array}}_{\alpha }\;, & \; & \sum_{\alpha \in I_{S}}\underset{s}{a}\;{\;}_{\alpha }^{\ell }{\begin{array}{c} A \end{array}}_{\alpha }\; & ⇌\sum_{\alpha \in I_{S}}\underset{s}{b}\;{\;}_{\alpha }^{\ell }{\begin{array}{c} A \end{array}}_{\alpha }\;, \end{array}$$

where ${\begin{array}{c} A \end{array}}_{\alpha }$ is used as a notation to refer to the different constituents. The constants $a_{\alpha }^{k}$, $b_{\alpha }^{k}$ are positive integers, and $\gamma_{\alpha }^{k}:=b_{\alpha }^{k}-a_{\alpha }^{k}$ denote the stoichiometric coefficients of the reactions. The net reaction rate is $R^{k}$, where reactions in the direction from left to right are counted as positive.

Each constituent has the (atomic) mass $m_{\alpha }$, for $\alpha \in I^{\pm }$ or $\alpha \in I_{S}$, and the net charge $z_{\alpha }e_{0}$, where $z_{\alpha }$ is the charge number of the constituent. Since charge and mass have to be conserved by each single reaction in the bulk and on the surface, we have

(42a) $$\begin{array}{cccccccc} \sum_{\alpha \in I^{\pm }}z_{\alpha }\gamma_{\alpha }^{k} & =0\;, & \; & \mathrm{and} & \; & \sum_{\alpha \in I^{\pm }}m_{\alpha }\gamma_{\alpha }^{k} & =0\;, & \end{array}$$

(42b) $$\begin{array}{ccccccc} \sum_{\alpha \in I_{S}}z_{\alpha }\underset{s}{\gamma_{\alpha }^{\ell }}\;\; & =0\;, & \; & \mathrm{and} & \; & \sum_{\alpha \in I_{S}}m_{\alpha }\underset{s}{\gamma_{\alpha }^{\ell }}\;\; & =0\;. \end{array}$$

To describe the thermodynamic state of the mixture, we use in the volume domains the inner energy density ρu, the particle number densities $n_{\alpha }$, and partial velocities $\upsilon_{\alpha }$ for $\alpha \in I^{\pm }$. On the surface, the surface inner energy density $\underset{s}{\rho }\;\;\underset{s}{u}\;\;$ and the number densities and partial velocities of the surface constituents are $\underset{s}{n}\;{\;}_{\alpha }$ and $\underset{s}{\upsilon }\;{\;}_{\alpha }$ for $\alpha \in I_{S}$. The multiplication of the number densities by $m_{\alpha }$ gives the partial mass densities:

(43) $$\begin{array}{cccc} \rho_{\alpha } & =m_{\alpha }n_{\alpha }\;, & \;\underset{s}{\rho }\;{\;}_{\alpha } & =m_{\alpha }\underset{s}{n}\;{\;}_{\alpha }\;. \end{array}$$

The mass density and the barycentric velocity of the mixture are defined by

(44a) $$\begin{array}{cccccc} \rho & =\sum_{\alpha \in I^{\pm }}\rho_{\alpha }\;, & \; & \upsilon & =\frac{1}{\rho }\sum_{\alpha \in I^{\pm }}\rho_{\alpha }\upsilon_{\alpha }\;, & \end{array}$$

(44b) $$\begin{array}{ccccc} \underset{s}{\rho }\;\; & =\sum_{\alpha \in I_{S}}\underset{s}{\rho }\;{\;}_{\alpha } & \; & \underset{s}{\upsilon }\;\; & =\frac{1}{\underset{s}{\rho }\;\;}\sum_{\alpha \in I_{S}}\underset{s}{\rho }\;{\;}_{\alpha }\underset{s}{\upsilon }\;{\;}_{\alpha }\;. \end{array}$$

The (non-convective) bulk and surface diffusion flux $J_{\alpha }$ and $\underset{s}{J}\;{\;}_{\alpha }$ with respect to the barycentric velocity are defined as

(45a) $$\begin{array}{cccc} J_{\alpha } & =\rho_{\alpha }\left(\upsilon_{\alpha }-\upsilon \right)\;,\; & \; & \;\;\mathrm{implying}\;\;\sum_{\alpha \in I^{\pm }}J_{\alpha }=0\;, \end{array}$$

(45b) $$\begin{array}{cccc} \underset{s}{J}\;{\;}_{\alpha } & =\underset{s}{\rho }\;{\;}_{\alpha }\left(\underset{s}{\upsilon }\;{\;}_{\alpha }-\underset{s}{\upsilon }\;\;\right)\;,\; & \; & \;\;\mathrm{implying}\;\;\sum_{\alpha \in I_{S}}\underset{s}{J}\;{\;}_{\alpha }=0\;. \end{array}$$

The free charge density and free current are are defined as

(46a) $$\begin{array}{cccccc} n^{F} & =e_{0}\sum_{\alpha \in I^{\pm }}z_{\alpha }n_{\alpha }\;, & \; & J^{F} & =e_{0}\sum_{\alpha \in I^{\pm }}z_{\alpha }J_{\alpha }\;, & \end{array}$$

(46b) $$\begin{array}{ccccc} \underset{s}{n}\;{\;}^{F} & =e_{0}\sum_{\alpha \in I_{S}}z_{\alpha }\underset{s}{n}\;{\;}_{\alpha }\;. & \; & \underset{s}{J}\;{\;}^{F} & =e_{0}\sum_{\alpha \in I_{S}}z_{\alpha }\underset{s}{J}\;{\;}_{\alpha }\;. \end{array}$$

### 4.2. Balance Equations

#### 4.2.1. Partial Mass Balances

In each of the subdomains $\Omega^{\pm }$, as well as on the surface *S*, the partial mass balances are

(47a) $$\begin{array}{cccc} \partial_{t}\rho_{\alpha }+\mathrm{div}\left(\rho_{\alpha }\upsilon +J_{\alpha }\right)\; & =\sum_{k}m_{\alpha }\gamma_{\alpha }^{k}R^{k} & \; & \;\mathrm{for}\;\alpha \in I^{\pm }\;, \end{array}$$

(47b) $$\begin{array}{cccc} \partial_{t,\nu }\underset{s}{\rho }\;{\;}_{\alpha }+{\left(\underset{s}{\rho }\;{\;}_{\alpha }\underset{s}{\upsilon }\;{\;}_{\tau }^{\Delta }+\underset{s}{J}\;{\;}_{\alpha ,\tau }^{\Delta }\right)}_{∥\Delta }-2k_{M}\underset{s}{\upsilon }\;{\;}_{\nu }\;\underset{s}{\rho }\;{\;}_{\alpha }\; & =\sum_{\ell }m_{\alpha }\underset{s}{\gamma }\;{\;}_{\alpha }^{\ell }\underset{s}{R}\;{\;}^{\ell }-\left[\;\left[\rho_{\alpha }\left(\upsilon_{\nu }-\underset{s}{\upsilon }\;{\;}_{\nu }\right)+J_{\alpha }\cdot \nu \right]\;\right] & \; & \;\mathrm{for}\;\alpha \in I_{S}\;. \end{array}$$

The partial mass balances can be combined to derive bulk and surface conservation laws for the total mass density of the mixture and for the free charge.

#### 4.2.2. Balance of Momentum

In the absence of electromagnetic fields, the momentum density of the mixture with respect to the barycentric velocity is ρυ. We postulate that, in the absence of gravitation, the total momentum of matter and the electromagnetic field is a conserved quantity. In the Galilean limits of the Maxwell equations, the Maxwell stress $\sigma^{\mathrm{EM}}$ equals the negative Lorentz force, while the electromagnetic momentum density is negligibly small. We introduce the *total stress tensor* Σ containing $\sigma^{\mathrm{EM}}$. Then, in either of the Galilean limit cases, the total momentum balances of the matter and electromagnetic field read in $\Omega^{\pm }$ and on *S*

(48a) $$\begin{array}{ccc} \partial_{t}\left(\rho \upsilon \right)+\mathrm{div}\left(\rho \upsilon ⊗\upsilon -\Sigma \right) & =\rho f\;, & \end{array}$$

(48b) $$\begin{array}{cc} \partial_{t,\nu }\left(\underset{s}{\rho }\;\;\underset{s}{\upsilon }\;{\;}^{i}\right)+{\left(\underset{s}{\rho }\;\;\underset{s}{\upsilon }\;{\;}^{i}\underset{s}{\upsilon }\;{\;}_{\tau }^{\Delta }-\underset{s}{\sigma }\;{\;}^{i\Delta }\right)}_{∥\Delta }-2k_{M}\underset{s}{\upsilon }\;{\;}_{\nu }\;\underset{s}{\rho }\;\;\underset{s}{\upsilon }\;{\;}^{i} & =\underset{s}{\rho }\;\;\underset{s}{f}\;{\;}^{i}-\left[\;\left[\rho \upsilon^{i}\left(\upsilon_{\nu }-\underset{s}{\upsilon }\;{\;}_{\nu }\right)-\Sigma^{ij}\nu_{j}\right]\;\right]\;, \end{array}$$

where f and $\underset{s}{f}\;\;$ are due to gravitation. The *surface momentum flux* $\underset{s}{\sigma }\;\;$ is decomposed into its normal and tangential components:

(49) $$\underset{s}{\sigma }\;{\;}^{i\Delta }=S^{\Gamma \Delta }\tau_{\Gamma }^{i}+S^{\Delta }\nu^{i}\;.$$

The tensor with the components $S^{\Gamma \Delta }$ is denoted as the *surface stress tensor*, and the vector with the components $S^{\Delta }$ is the *normal stress vector*. We neglect internal spin. Given the symmetry of the Maxwell stress tensor due to the Maxwell–Lorentz aether relations, this implies the symmetry of the total stress tensors and the vanishing of the normal surface stress [10], i.e., the assumption implies, for i,j=1,2,3 and Γ,Δ=1,2,

(50) $$\Sigma^{ij}=\Sigma^{ji}\;,\;S^{\Gamma \Delta }=S^{\Delta \Gamma }\;\;\mathrm{and}\;\;S^{\Delta }=0\;.$$

From these balances, several different balances for the momentum ρυ can be derived, depending on which forces should be accounted for on the right-hand side. Subtracting from (48) the momentum balance (30) in the quasi-electrostatic limit, or (36) in the quasi-magnetostatic limit, then yields the momentum balance equations of matter with the Lorentz force k, respectively $\underset{s}{k}\;\;$, on the right-hand side. In addition to the Lorentz force, one might also want to track the *Kelvin polarisation force, electrostrictive force*, or *Korteweg–Helmholtz force* explicitly in the balances (cf. Ref. [7] ), such that the stress tensor then needs to be modified accordingly.

#### 4.2.3. Balance of Inner Energy

The energy of matter consists of the *inner energy* density ρu and the *kinetic energy* density ${\rho |\upsilon |}^{2}$. The balance equations of the mass and momentum of matter imply the balances of the kinetic energy in $\Omega^{\pm }$ and on *S*. Moreover, we postulate that, in the absence of gravitation, the *total energy* of the matter and electromagnetic field is a conserved quantity. This implies the inner energy balances in $\Omega^{\pm }$ and on *S* as shown in Appendix A, viz.

(51a) $$\begin{array}{ccc} \partial_{t}\rho u+\mathrm{div}\left(\rho u\;\upsilon +Q\right)= & \;\pi -k\cdot \upsilon +\mathrm{div}\left(E\times M\right)\;+\left(\Sigma -\sigma^{\mathrm{EM}}\right):\nabla \upsilon \;. & \end{array}$$

(51b) $$\begin{array}{cc} \partial_{t,\nu }\underset{s}{\rho }\;\;\underset{s}{u}\;\;+{\left(\underset{s}{\rho }\;\;\underset{s}{u}\;\;)\;\underset{s}{\upsilon }\;{\;}_{\tau }^{\Delta }+\underset{s}{q}\;{\;}^{\Delta }\right)}_{∥\Delta }-2k_{M}\underset{s}{\upsilon }\;{\;}_{\nu }\;\underset{s}{\rho }\;\;\underset{s}{u}\;\;= & \;\underset{s}{\pi }\;\;-\underset{s}{k}\;\;\cdot \underset{s}{\upsilon }\;\;+\left[\;\left[\left(E\times M\right)\cdot \nu \right]\;\right]\;+\underset{s}{\sigma }\;{\;}^{i\Delta }\;\underset{s}{\upsilon }\;{\;}_{∥\Delta }^{i}\\ \; & -\left[\;\;\left[\rho u\;(\upsilon_{\nu }-\underset{s}{\upsilon }\;{\;}_{\nu })+Q\cdot \nu \right]\;\;\right]\\ \; & -\left[\;\;\left[\frac{1}{2}\rho {\left|\upsilon -\underset{s}{\upsilon }\;\;\right|}^{2}\left(\upsilon_{\nu }-\underset{s}{\upsilon }\;{\;}_{\nu }\right)-\left(\Sigma -\sigma^{\mathrm{EM}}\right)\left(\upsilon -\underset{s}{\upsilon }\;\;\right)\cdot \nu \right]\;\;\right]\;. \end{array}$$

We can write Q=q+E×M, such that q coincides with the heat flux in the balance of the inner and kinetic energy when the heat production is given by the Joule heat. Using the particular form of the Lorentz force and the Joule heat in the quasi-electrostatic limit, we obtain for the inner energy balance

$$\begin{array}{cc} \partial_{t}\rho u+\mathrm{div}\left(\rho u\;\upsilon +Q\right)= & \;J^{F}\cdot E+\left(\Sigma -\sigma^{\mathrm{EM}}-E⊗P+\left(E\cdot P\right)1\right):\nabla \upsilon \end{array}$$

(52a) $$\begin{array}{cc} \; & +\left(\partial_{t}P+\left(\upsilon \cdot \nabla \right)P\right)\cdot E\;, \end{array}$$

(52b) $$\begin{array}{cc} \partial_{t,\nu }\left(\underset{s}{\rho }\;\;\underset{s}{u}\;\;\right)+{\left(\underset{s}{\rho }\;\;\underset{s}{u}\;\;\;\underset{s}{\upsilon }\;{\;}_{\tau }^{\Delta }+\underset{s}{q}\;{\;}^{\Delta }\right)}_{∥\Delta }-2k_{M}\underset{s}{\upsilon }\;{\;}_{\nu }\;\underset{s}{\rho }\;\;\underset{s}{u}\;\;= & \;\underset{s}{J}\;{\;}^{F}\cdot \bar{E}\;+\underset{s}{\sigma }\;{\;}^{i\Delta }\;\underset{s}{\upsilon }\;{\;}_{∥\Delta }^{i}\\ \; & -\left[\;\;\left[\left(\rho u+\frac{1}{2}\rho {\left|\upsilon -\underset{s}{\upsilon }\;\;\right|}^{2}\right)\;\left(\upsilon_{\nu }-\underset{s}{\upsilon }\;{\;}_{\nu }\right)+Q\cdot \nu \right]\;\;\right]\\ \; & +\left[\;\;\left[\left(\upsilon -\underset{s}{\upsilon }\;\;\right)\cdot \left(\Sigma -\sigma^{\mathrm{EM}}-E⊗P+\left(E\cdot P\right)\;1\right)\cdot \nu \right]\;\;\right]\;. \end{array}$$

On the other hand, we obtain in the quasi-magnetostatic limit from the explicit Lorentz force and Joule heat

$$\begin{array}{cc} \partial_{t}\rho u+\mathrm{div}\left(\rho u\;\upsilon +Q\right)= & \;J^{F}\cdot \left(\upsilon \times B\right)+\left(\Sigma -\sigma^{\mathrm{EM}}+M⊗B-\left(M\cdot B\right)1\right):\nabla \upsilon \end{array}$$

(53a) $$\begin{array}{cc} \; & -\left(\partial_{t}B+\left(\upsilon \cdot \nabla \right)B\right)\cdot M\;, \end{array}$$

(53b) $$\begin{array}{cc} \partial_{t,\nu }\left(\underset{s}{\rho }\;\;\underset{s}{u}\;\;\right)+{\left(\underset{s}{\rho }\;\;\underset{s}{u}\;\;\;\underset{s}{\upsilon }\;{\;}_{\tau }^{\Delta }+\underset{s}{q}\;{\;}^{\Delta }\right)}_{∥\Delta }-2k_{M}\underset{s}{\upsilon }\;{\;}_{\nu }\;\underset{s}{\rho }\;\;\underset{s}{u}\;\;= & \;\underset{s}{J}\;{\;}^{F}\cdot \left(\underset{s}{\upsilon }\;\;\times \bar{B}\right)\;+\underset{s}{\sigma }\;{\;}^{i\Delta }\;\underset{s}{\upsilon }\;{\;}_{∥\Delta }^{i}\\ \; & -\left[\;\;\left[\left(\rho u+\frac{1}{2}\rho {\left|\upsilon -\underset{s}{\upsilon }\;\;\right|}^{2}\right)\;\left(\upsilon_{\nu }-\underset{s}{\upsilon }\;{\;}_{\nu }\right)+Q\cdot \nu \right]\;\;\right]\\ \; & +\left[\;\;\left[\left(\upsilon -\underset{s}{\upsilon }\;\;\right)\cdot \left(\Sigma -\sigma^{\mathrm{EM}}+M⊗B-\left(M\cdot B\right)\;1\right)\cdot \nu \right]\;\;\right]\;. \end{array}$$

## 5. Constitutive Equations for the Quasi-Electrostatic Limit

In the quasi-electrostatic limit, the coupled electrothermodynamic system consists of (29), (47), (48), and (52). To close the coupled system, additional constitutive relations are needed. These can be obtained from the application of an entropy principle as described in [8]. Because it allows a straightforward derivation of the entropy production in the desired form, we assume constitutive functions of the entropy densities of the form:

(54) $$\begin{array}{cccc} \rho \eta & =\rho \tilde{\eta }\left(\rho u,{\left(\rho_{\alpha }\right)}_{\alpha \in I^{\pm }},P\right)\;, & \underset{s}{\rho }\;\;\underset{s}{\eta }\;\; & =\underset{s}{\rho }\;\;\underset{s}{\tilde{\eta }}\;\;\left(\underset{s}{\rho }\;\;\underset{s}{u}\;\;,{\left(\underset{s}{\rho }\;{\;}_{\alpha }\right)}_{\alpha \in I_{S}}\right)\;. \end{array}$$

In principle, it would also be possible to include the dependence of $\rho \tilde{\eta }$ on M, such that, by the application of the entropy principle, one more constitutive equation could be obtained, which then would allow solving (33) and evaluating the electric current $j^{e}$. In the quasi-electrostatic limit, however, all explicit dependence of the system (29), (47), (48), and (52) on B and M is removed due to the asymptotic smallness of those terms. The application of the entropy principle builds on this system of equations, and therefore, it should not reintroduce asymptotically non-negligible dependencies on B and M. Moreover, the terms in the entropy production depending on B and M would be in the same asymptotic order as the terms that have been neglected in the system (29), (47), (48), and (52). Therefore, the value of the constitutive equations based on such small terms in the entropy production must be questionable. Instead, it seems more advisable to use a constitutive equation relating B and M, which were derived from another source, which might well be the complementary quasi-magnetostatic limit in Section 6.

The (absolute) temperature $T,\underset{s}{T}\;\;$ and the chemical potentials $\mu_{\alpha },\underset{s}{\mu }\;{\;}_{\alpha }$ are defined as

(55a) $$\begin{array}{ccccc} \frac{1}{T} & =\frac{\partial \rho \tilde{\eta }}{\partial \rho u}\;, & \; & \frac{\mu_{\alpha }}{T} & =-\frac{\partial \rho \tilde{\eta }}{\partial \rho_{\alpha }}\;, \end{array}$$

(55b) $$\begin{array}{ccccc} \frac{1}{\underset{s}{T}\;\;} & =\frac{\partial \underset{s}{\rho }\;\;\underset{s}{\tilde{\eta }}\;\;}{\partial \underset{s}{\rho }\;\;\underset{s}{u}\;\;}\;, & \; & \frac{\underset{s}{\mu }\;{\;}_{\alpha }}{\underset{s}{T}\;\;} & =-\frac{\partial \underset{s}{\rho }\;\;\underset{s}{\tilde{\eta }}\;\;}{\partial \underset{s}{\rho }\;{\;}_{\alpha }}\;. \end{array}$$

For the construction of the constitutive equations, it is often beneficial to use the temperature *T*, respectively $\underset{s}{T}\;\;$, as an independent variable, instead of the inner energy density. We, thus, introduce the *free energy* densities ρψ and $\underset{s}{\rho }\;\;\underset{s}{\psi }\;\;$ by means of the Legendre transformation of the entropy density ρη, viz.

(56) $$\begin{array}{ccccc} \rho \psi & =\rho u-T\;\rho \eta \;, & \; & \underset{s}{\rho }\;\;\underset{s}{\psi }\;\; & =\underset{s}{\rho }\;\;\underset{s}{u}\;\;-\underset{s}{T}\;\;\;\underset{s}{\rho }\;\;\underset{s}{\eta }\;\;\;. \end{array}$$

For the free energy density and the entropy density, we, thus, have representations as functions of the temperature as

(57) $$\begin{array}{ccccc} \rho \psi & =\rho \overset{ˇ}{\psi }\left(T,{\left(\rho_{\alpha }\right)}_{\alpha \in I^{\pm }},P\right)\;, & \; & \underset{s}{\rho }\;\;\underset{s}{\psi }\;\; & =\underset{s}{\rho }\;\;\underset{s}{\hat{\psi }}\;\;\left(\underset{s}{T}\;\;,{\left(\underset{s}{\rho }\;{\;}_{\alpha }\right)}_{\alpha \in I_{S}}\right)\;, \end{array}$$

and in an analogous way for the inner energy and entropy. From the construction (56) and the definition of the temperature according to (55), we obtain the well-known thermodynamic relations:

(58a) $$\begin{array}{cccccc} \frac{\partial \rho \overset{ˇ}{\psi }}{\partial T} & =-\rho \overset{ˇ}{\eta }\;, & \; & \frac{\partial }{\partial T}\left(\frac{\rho \overset{ˇ}{\psi }}{T}\right) & =-\frac{\rho \overset{ˇ}{u}}{T^{2}}\;, & \end{array}$$

(58b) $$\begin{array}{ccccc} \frac{\partial \underset{s}{\rho }\;\;\underset{s}{\hat{\psi }}\;\;}{\partial \underset{s}{T}\;\;} & =-\underset{s}{\rho }\;\;\underset{s}{\hat{\eta }}\;\;\;, & \; & \frac{\partial }{\partial \underset{s}{T}\;\;}\left(\frac{\underset{s}{\rho }\;\;\underset{s}{\hat{\psi }}\;\;}{\underset{s}{T}\;\;}\right) & =-\frac{\underset{s}{\rho }\;\;\underset{s}{\hat{u}}\;\;}{\underset{s}{T}\;{\;}^{2}}\;. \end{array}$$

### 5.1. Constitutive Relations for the Bulk

To determine the entropy production in a straightforward manner, the chain rule is applied to the time derivative of $\rho \tilde{\eta }$, and the partial mass balances (47) and the inner energy balance (52) are applied. Then, the choice of the entropy flux fixes the entropy production. Nevertheless, the entropy production can still be rewritten in such a way that is most suitable for the derivation of the constitutive relations. We choose the entropy flux as $\phi =\frac{Q}{T}-\sum_{\alpha \in I}\frac{\mu_{\alpha }}{T}J_{\alpha }$. Moreover, we define the viscous stress tensor and the symmetric velocity gradient as

(59a) $$\begin{array}{cc} T=\; & \Sigma -\sigma^{\mathrm{EM}}-\left(\rho u-T\rho \tilde{\eta }-E\cdot P-\sum_{\alpha \in I\pm }\rho_{\alpha }\mu_{\alpha }\right)1-\frac{1}{2}\left(E⊗P+P⊗E\right)\;, \end{array}$$

(59b) $$\begin{array}{cc} D=\; & \frac{1}{2}\left(\nabla \upsilon +\nabla \upsilon^{T}\right)\;. \end{array}$$

To account in the derivation of the entropy production for the constraint (45) on the mass fluxes, we choose in each subdomain one designated constituent as a reference and denote these constituents by $A_{0-}$, $A_{0+}$, respectively. Moreover, we use a symmetry condition originating from the transformation properties of the thermodynamic fields, cf. Ref. [8]. Then, we obtain the entropy production as the sum of binary products, viz.

(60) $$\begin{array}{cc} 0\overset{!}{\leq }\xi =\;\; & \underset{=\xi_{SV}}{\underset{︸}{\frac{1}{T}\left(T-\frac{1}{3}\mathrm{trace}\left(T\right)1\right):\left(D-\frac{1}{3}\mathrm{trace}\left(D\right)1\right)}}\;\;+\underset{\xi_{VV}}{\underset{︸}{\frac{1}{3}\frac{1}{T}\mathrm{trace}\left(T\right)\cdot \mathrm{trace}\left(D\right)}}\\ \; & +\underset{=\xi_{TD}}{\underset{︸}{Q\cdot \nabla \;\left(\;\frac{1}{T}\;\right)\;-\;\sum_{\alpha \in I^{\pm }\setminus \left\{A_{0\pm }\right\}}J_{\alpha }\cdot \left(\nabla \left(\frac{\mu_{\alpha }}{T}-\frac{\mu_{0\pm }}{T}\right)-\frac{1}{T}\;\left(\frac{z_{\alpha }e_{0}}{m_{\alpha }}-\frac{z_{0\pm }e_{0}}{m_{0\pm }}\right)E\right)}}\\ \; & +\underset{\xi_{R}}{\underset{︸}{\frac{1}{T}\sum_{k}\left(-\sum_{\alpha \in I^{\pm }}\gamma_{\alpha }^{k}\;m_{\alpha }\mu_{\alpha }\right)\cdot R^{k}}}\;+\underset{\xi_{P}}{\underset{︸}{\left(\frac{\partial \rho \tilde{\eta }}{\partial P}+\frac{1}{T}E\right)\cdot \left(\partial_{t}P+\left(\upsilon \cdot \nabla \right)P-\frac{1}{2}\left(\nabla \upsilon -\nabla \upsilon^{T}\right)P\right)}}\;. \end{array}$$

We now can identify the dissipation mechanisms related to their specific entropy production. To reduce the complexity of the constitutive equations, we neglect cross-effects other than thermodiffusion. Thus, we consider the five dissipation mechanisms: shear viscosity $\xi_{SV}$, volume viscosity $\xi_{VV}$, (bulk-)reactions $\xi_{R}$, thermodiffusion $\xi_{TD}$, and polarisation $\xi_{P}$. For each dissipation mechanism, we apply closure relations to obtain the constitutive equations.

#### 5.1.1. Thermodiffusion

For the heat flux and the mass fluxes, we choose a linear relation with cross-effects. We chose coefficients κ, $L_{\beta }$, $M_{\alpha \beta }$ such that the coefficient matrix is symmetric and positive definite. In particular, the heat conductivity κ and the mobility matrix *M* are symmetric and positive definite. We set for $\alpha \in I^{\pm }\setminus \left\{A_{0\pm }\right\}$

(61a) $$\begin{array}{cc} Q & =-\frac{\kappa }{T^{2}}\nabla T\;-\sum_{\beta \in I^{\pm }\setminus \left\{A_{0\pm }\right\}}L_{\beta }\left(\nabla \left(\frac{\mu_{\beta }}{T}-\frac{\mu_{0\pm }}{T}\right)-\frac{1}{T}\left(\frac{z_{\beta }e_{0}}{m_{\beta }}-\frac{z_{0\pm }e_{0}}{m_{0\pm }}\right)E\right)\;, \end{array}$$

(61b) $$\begin{array}{cc} J_{\alpha } & =-\frac{L_{\alpha }}{T^{2}}\nabla T\;-\;\;\sum_{\beta \in I^{\pm }\setminus \left\{A_{0\pm }\right\}}\;\;\;M_{\alpha \beta }\left(\nabla \left(\frac{\mu_{\beta }}{T}-\frac{\mu_{0\pm }}{T}\right)-\frac{1}{T}\left(\frac{z_{\beta }e_{0}}{m_{\beta }}-\frac{z_{0\pm }e_{0}}{m_{0\pm }}\right)E\right)\;. \end{array}$$

#### 5.1.2. Reactions

For simplicity, we neglect cross-effects between the different chemical reactions. We choose positive coefficients $A^{k},R_{0}^{k}$ and and apply a nonlinear closure relation to obtain, for any of the reactions in the volume domains,

(62) $$\begin{array}{cc} R^{k} & =R_{0}^{k}\;\left(1-\exp\left(\frac{A^{k}}{k_{B}T}\sum_{\alpha \in I^{\pm }}\gamma_{\alpha }^{k}m_{\alpha }\mu_{\alpha }\right)\right)\;. \end{array}$$

#### 5.1.3. Polarisation

We chose a linear closure relation with the relaxation time $\tau^{P}\geq 0$ of polarisation to obtain

(63) $$\begin{array}{cc} E+T\frac{\partial \rho \tilde{\eta }}{\partial P}\; & =\frac{\tau^{P}}{\varepsilon_{0}}\;\left(\partial_{t}P+\left(\upsilon \cdot \nabla \right)P-\frac{1}{2}\left(\nabla \upsilon -\nabla \upsilon^{T}\right)P\right)\;. \end{array}$$

#### 5.1.4. Viscosity

Linear relations for the volume viscosity and for the shear viscosity with phenomenological coefficients $\eta_{b}$, $\eta_{s}$ that satisfy $(\eta_{b}+\frac{2}{3}\eta_{s})>0$ and $\eta_{s}>0$ yield

(64) $$\begin{array}{cc} T & =\eta_{b}\mathrm{div}\left(\upsilon \right)1+\eta_{s}\left(\nabla \upsilon +{\left(\nabla \upsilon \right)}^{T}\right)\;. \end{array}$$

This implies the constitutive equation for the symmetric stress tensor:

(65) $$\begin{array}{cc} \Sigma -\sigma^{\mathrm{EM}}\; & =T+\left(\rho u-T\rho \tilde{\eta }-E\cdot P-\sum_{\alpha \in I^{\pm }}\rho_{\alpha }\mu_{\alpha }\right)\;1+\frac{1}{2}\left(E⊗P+P⊗E\right)\;. \end{array}$$

### 5.2. Constitutive Relations for the Surface

We proceed in analogous manner as in the volume and choose the surface entropy flux as $\underset{s}{\phi }\;\;=\frac{\underset{s}{q}\;\;}{\underset{s}{T}\;\;}-\sum_{\alpha \in I_{S}}\frac{\underset{s}{\mu }\;{\;}_{\alpha }}{\underset{s}{T}\;\;}\underset{s}{J}\;{\;}_{\alpha }$. We define the tensors T and D with the components:

(66a) $$\begin{array}{cc} \underset{s}{T}\;{\;}^{\Delta \Gamma } & =S^{\Gamma \Delta }-\left(\underset{s}{\rho }\;\;\underset{s}{u}\;\;-\underset{s}{T}\;\;\underset{s}{\rho }\;\;\underset{s}{\tilde{\eta }}\;\;-\sum_{\alpha \in I_{S}}\underset{s}{\mu }\;{\;}_{\alpha }\underset{s}{\rho }\;{\;}_{\alpha }\right)g^{\Delta \Gamma }\;, \end{array}$$

(66b) $$\begin{array}{cc} \underset{s}{D}\;{\;}_{\Delta \Gamma } & =\frac{1}{2}\left(g_{\Gamma \Lambda }\;\underset{s}{\upsilon }\;{\;}_{\tau ∥\Delta }^{\Lambda }+g_{\Delta \Lambda }\;\underset{s}{\upsilon }\;{\;}_{\tau ∥\Gamma }^{\Lambda }\right)-b_{\Gamma \Delta }\;\underset{s}{\upsilon }\;{\;}_{\nu }\;. \end{array}$$

Moreover, we choose one designated constituent $A_{0}\in I_{S}$ on the surface. Then, we obtain the surface entropy production as a sum of binary products, viz.

(67) $$\begin{array}{cc} 0\overset{!}{\leq }\underset{s}{\xi }\;\;=\;\; & \underset{=\underset{s}{\xi }\;{\;}_{V}^{\tau }}{\underset{︸}{\frac{1}{\underset{s}{T}\;\;}\left[\underset{s}{T}\;{\;}^{\Delta \Gamma }\right]\cdot \left(\underset{s}{D}\;{\;}_{\Delta \Gamma }\right)}}\;\underset{\underset{s}{\xi }\;{\;}_{R}}{\underset{︸}{-\frac{1}{\underset{s}{T}\;\;}\sum_{\ell }\left(\sum_{\beta \in I_{S}}\underset{s}{\gamma }\;{\;}_{\beta }^{\ell }\;m_{\beta }\underset{s}{\mu }\;{\;}_{\beta }\right)\cdot \underset{s}{R}\;{\;}^{\ell }}}\\ \; & \underset{=\underset{s}{\xi }\;{\;}_{TD}^{\tau }}{\underset{︸}{+\underset{s}{q}\;{\;}^{\Delta }\cdot {\left(\frac{1}{\underset{s}{T}\;\;}\right)}_{∥\Delta }-\sum_{\alpha \in I_{S}\setminus \left\{A_{0}\right\}}\underset{s}{J}\;{\;}_{\alpha ,\tau }^{\Delta }\cdot \left({\left(\frac{\underset{s}{\mu }\;{\;}_{\alpha }}{\underset{s}{T}\;\;}-\frac{\underset{s}{\mu }\;{\;}_{0}}{\underset{s}{T}\;\;}\right)}_{∥\Delta }-\frac{1}{\underset{s}{T}\;\;}\left(\frac{z_{\alpha }e_{0}}{m_{\alpha }}-\frac{z_{0}e_{0}}{m_{0}}\right)g_{\Delta \Gamma }{\bar{E}}_{\tau }^{\Gamma }\right)}}\\ \; & \underset{=\underset{s}{\xi }\;{\;}_{H}^{\nu }}{\underset{︸}{+\left[\;\;\left[\left(Q_{\nu }+\left(T\rho \tilde{\eta }+\sum_{\alpha \in I^{\pm }}\rho_{\alpha }\mu_{\alpha }\right)\left(\upsilon_{\nu }-\underset{s}{\upsilon }\;{\;}_{\nu }\right)\right)\cdot \left(\frac{1}{T}-\frac{1}{\underset{s}{T}\;\;}\right)\right]\;\;\right]}}\\ \; & \underset{=\underset{s}{\xi }\;{\;}_{V}^{\nu }}{\underset{︸}{+\frac{1}{\underset{s}{T}\;\;}\left[\;\;\left[\left(\upsilon -\underset{s}{\upsilon }\;\;\right)\cdot \left(T-\frac{1}{2}\left(E⊗P-P⊗E\right)-\left(\frac{1}{2}\rho {\left|\underset{s}{\upsilon }\;\;-\upsilon \right|}^{2}+\underset{s}{T}\;\;\rho \left(\frac{\mu_{0\pm }}{T}-\frac{\underset{s}{\mu }\;{\;}_{0\pm }}{\underset{s}{T}\;\;}\right)\right)1\right)\nu \right]\;\;\right]}}\\ \; & \underset{=\underset{s}{\xi }\;{\;}_{MT}^{\nu }}{\underset{︸}{-\left[\;\;\left[\sum_{\alpha \in I^{\pm }\setminus \left\{A_{0\pm }\right\}}\left(J_{\alpha ,\nu }+\rho_{\alpha }\left(\upsilon_{\nu }-\underset{s}{\upsilon }\;{\;}_{\nu }\right)\right)\cdot \left(\frac{\mu_{\alpha }-\mu_{0\pm }}{T}-\frac{\underset{s}{\mu }\;{\;}_{\alpha }-\underset{s}{\mu }\;{\;}_{0\pm }}{\underset{s}{T}\;\;}\right)\right]\;\;\right]}}\;. \end{array}$$

We identify six dissipation mechanisms: tangential surface viscosity $\underset{s}{\xi }\;{\;}_{V}^{\tau }$, tangential surface thermodiffusion $\underset{s}{\xi }\;{\;}_{TD}^{\tau }$, surface reactions $\underset{s}{\xi }\;{\;}_{R}$, heat transport normal to the surface $\underset{s}{\xi }\;{\;}_{H}^{\nu }$, mass transport normal to the surface $\underset{s}{\xi }\;{\;}_{MT}^{\nu }$, and viscosity normal to the surface $\underset{s}{\xi }\;{\;}_{V}^{\nu }$. Again, cross-effects other than the thermodiffusion were neglected.

#### 5.2.1. Thermodiffusion

We chose phenomenological coefficients $\underset{s}{\kappa }\;\;$, $\underset{s}{L}\;{\;}_{\beta }$, and $\underset{s}{M}\;{\;}_{\alpha \beta }$, such that the coefficient matrix is symmetric and positive definite. We set, for $\alpha \in I_{S}\setminus \left\{A_{0}\right\}$,

(68a) $$\begin{array}{ccc} \underset{s}{q}\;{\;}_{\tau }^{\Delta }=\; & -\frac{\underset{s}{\kappa }\;\;}{\underset{s}{T}\;{\;}^{2}}g^{\Delta \Gamma }\underset{s}{T}\;{\;}_{∥\Gamma } & -\sum_{\beta \in I_{S}\setminus \left\{A_{0}\right\}}\underset{s}{L}\;{\;}_{\beta }\;\left[g^{\Delta \Gamma }{\left(\frac{\underset{s}{\mu }\;{\;}_{\beta }}{\underset{s}{T}\;\;}-\frac{\underset{s}{\mu }\;{\;}_{0}}{\underset{s}{T}\;\;}\right)}_{∥\Gamma }-\frac{1}{\underset{s}{T}\;\;}\left(\frac{z_{\beta }e_{0}}{m_{\beta }}-\frac{z_{0}e_{0}}{m_{0}}\right){\bar{E}}_{\tau }^{\Delta }\right]\;, \end{array}$$

(68b) $$\begin{array}{ccc} \underset{s}{J}\;{\;}_{\alpha ,\tau }^{\Delta }=\; & -\frac{\underset{s}{L}\;{\;}_{\alpha }}{\underset{s}{T}\;{\;}^{2}}g^{\Delta \Gamma }\underset{s}{T}\;{\;}_{∥\Gamma } & -\sum_{\beta \in I_{S}\setminus \left\{A_{0}\right\}}\;\;\underset{s}{M}\;{\;}_{\alpha \beta }\;\left[g^{\Delta \Gamma }{\left(\frac{\underset{s}{\mu }\;{\;}_{\beta }}{\underset{s}{T}\;\;}-\frac{\underset{s}{\mu }\;{\;}_{0}}{\underset{s}{T}\;\;}\right)}_{∥\Gamma }-\frac{1}{\underset{s}{T}\;\;}\left(\frac{z_{\beta }e_{0}}{m_{\beta }}-\frac{z_{0}e_{0}}{m_{0}}\right){\bar{E}}_{\tau }^{\Delta }\right]\;. \end{array}$$

#### 5.2.2. Surface Reactions

We neglect cross-effects between the different reactions and apply the nonlinear closure relations with positive phenomenological coefficients $\underset{s}{A}\;{\;}^{\ell },\underset{s}{R}\;{\;}_{0}^{\ell }$:

(69) $$\begin{array}{cc} \underset{s}{R}\;{\;}^{\ell } & =\underset{s}{R}\;{\;}_{0}^{\ell }\;\left(1-\exp\left(\frac{\underset{s}{A}\;{\;}^{\ell }}{k_{B}\underset{s}{T}\;\;}\sum_{\alpha \in I_{S}}\underset{s}{\gamma }\;{\;}_{\alpha }^{\ell }m_{\alpha }\underset{s}{\mu }\;{\;}_{\alpha }\right)\right)\;. \end{array}$$

#### 5.2.3. Surface Viscosity

Linear closure for the trace and for the deviatoric part of the surface stress tensor $\underset{s}{T}\;\;$ with phenomenological coefficients satisfying $\underset{s}{\eta }\;{\;}_{b}+\underset{s}{\eta }\;{\;}_{s}\geq 0$ and $\underset{s}{\eta }\;{\;}_{s}\geq 0$ yields the constitutive equation:

(70) $$\begin{array}{cc} \underset{s}{T}\;\;\; & =\underset{s}{\eta }\;{\;}_{b}\;\mathrm{trace}\left(\underset{s}{D}\;\;\;g^{-1}\right)\;g^{-1}+2\underset{s}{\eta }\;{\;}_{s}\;g^{-1}\underset{s}{D}\;\;\;g^{-1}\;. \end{array}$$

This implies, for the symmetric surface stress tensor, the constitutive equation:

(71) $$\begin{array}{cc} S^{\Gamma \Delta }= & \left(\underset{s}{\rho }\;\;\underset{s}{u}\;\;-\underset{s}{T}\;\;\underset{s}{\rho }\;\;\underset{s}{\tilde{\eta }}\;\;-\sum_{\alpha \in I_{S}}\underset{s}{\mu }\;{\;}_{\alpha }\underset{s}{\rho }\;{\;}_{\alpha }\right)g^{\Delta \Gamma }+\underset{s}{T}\;{\;}^{\Gamma \Delta }\;. \end{array}$$

#### 5.2.4. Heat Flux Normal to the Surface

We choose coefficients $\underset{s}{\kappa }\;{\;}^{\pm }$ and obtain from the linear closure:

(72) $$\begin{array}{cc} {\left(Q_{\nu }+\left(T\rho \tilde{\eta }+\sum_{\alpha \in I^{\pm }}\rho_{\alpha }\mu_{\alpha }\right)\left(\upsilon_{\nu }-\underset{s}{\upsilon }\;{\;}_{\nu }\right)\right)}^{\pm }\; & =\pm \underset{s}{\kappa }\;{\;}^{\pm }\;{\left(\frac{1}{T}-\frac{1}{\underset{s}{T}\;\;}\right)}^{\pm }\;. \end{array}$$

#### 5.2.5. Stress from the Volume

We choose coefficients $\underset{s}{\lambda }\;{\;}^{\pm }$ and $\underset{s}{\eta }\;{\;}_{\Delta }^{\pm }>0$, for Δ=1,2, and obtain from the linear closure:

(73a) $$\begin{array}{cc} {\left(\nu \cdot \left(T-\frac{1}{2}\left(E⊗P-P⊗E\right)\right)\tau_{\Gamma }\;g^{\Gamma \Delta }\right)}^{\pm }\; & =\pm \underset{s}{\eta }\;{\;}_{\Delta }^{\pm }\;{\left(\upsilon_{\tau }^{\Delta }-\underset{s}{\upsilon }\;{\;}_{\tau }^{\Delta }\right)}^{\pm }\;. \end{array}$$

(73b) $$\begin{array}{cc} (\nu \cdot \left(T-\frac{1}{2}\left(E⊗P-P⊗E\right)\right)\nu -(\frac{1}{2}\rho {\left|\underset{s}{\upsilon }\;\;-\upsilon \right|}^{2}+\underset{s}{T}\;\;\rho \;{\left(\frac{\mu_{0\pm }}{T}-\frac{\underset{s}{\mu }\;{\;}_{0\pm }}{\underset{s}{T}\;\;}))\right)}^{\pm }\; & =\pm \underset{s}{\lambda }\;{\;}^{\pm }\rho^{\pm }\;{\left(\rho \;(\upsilon_{\nu }-\underset{s}{\upsilon }\;{\;}_{\nu })\right)}^{\pm }\;. \end{array}$$

#### 5.2.6. Mass Transport Normal to the Surface

The mass transport normal to the surface can be related to the adsorption, which, in the context of electrothermodynamics, is formally different from the mass production due to chemical reactions. However, in experiments, it might not always be clear how to distinguish between adsorption and surface reactions, and we, therefore, here, also applied a nonlinear closure similar to the chemical reactions. We neglect cross-effects and chose for $\alpha \in I^{\pm }\setminus \left\{A_{0\pm }\right\}$ coefficients $\beta_{\alpha }^{\pm }$ and $\underset{s}{M}\;{\;}_{\alpha }^{\pm }>0$ to obtain, for $\alpha \in I^{\pm }\setminus \left\{A_{0\pm }\right\}$,

(74a) $$\begin{array}{ccc} D_{\alpha }^{\pm }\; & ={\left(\mu_{\alpha }-\mu_{0\pm }-\frac{T}{\underset{s}{T}\;\;}\left(\underset{s}{\mu }\;{\;}_{\alpha }-\underset{s}{\mu }\;{\;}_{0\pm }\right)\right)}^{\pm }\;, & \end{array}$$

(74b) $$\begin{array}{ccc} {\left(J_{\alpha ,\nu }+\rho_{\alpha }\left(\upsilon_{\nu }-\underset{s}{\upsilon }\;{\;}_{\nu }\right)\right)}^{\pm }\; & =\mp \underset{s}{M}\;{\;}_{\alpha }^{\pm }\left(\exp\left(\frac{\beta_{\alpha }^{\pm }}{k_{B}T}D_{\alpha }^{\pm }\right)-\exp\left(\frac{\beta_{\alpha }^{\pm }-1}{k_{B}T}D_{\alpha }^{\pm }\right)\right)\;. & \end{array}$$

### 5.3. Discussion of a General Free Energy Model

The obtained constitutive equations are general, in the sense that they were derived without making use of any particular specific material properties. They only rely on the universal balance equations and the entropy principle. All material properties of a specific electrochemical system, thus, have to be incorporated into the constitutive functions of the entropy and the phenomenological coefficients. We now restrict the constitutive function for the free energy to the structure:

(75) $$\begin{array}{c} \rho \psi =\hat{\rho \psi }\left(T,{\left(\rho_{\alpha }\right)}_{\alpha \in I}\right)+\rho {\overset{ˇ}{\psi }}^{\mathrm{pol},P}(T,{\left(\rho_{\alpha }\right)}_{\alpha \in I},|P|)\;, \end{array}$$

where the first part is independent of the electromagnetic field and the second part depends only on the absolute value of P. Different free energy models can be used for the field-independent free energy contribution $\rho \hat{\psi }$, depending on the considered material. For liquid electrolytes, the model of simple mixtures of solvated ions (cf. [5,17]) appears to be appropriate. The general construction of suitable free energies and their incompressible limit is analysed in [18]. The analysis emphasizes the importance of linear approaches with respect to the partial molar volume and illustrates the possibility of recovering the nonlinear behaviour of the mixtures based on the nonlinear reaction behaviour. As an example of a different material model, we refer to [19], which models yttria-stabilised zirconia.

#### 5.3.1. Dielectric Susceptibility and Debye Relaxation

Since it might appear more familiar to formulate constitutional equations in terms of E instead of P, we introduce a conjugate variable to P which coincides with E in the case of polarisation equilibrium. We define

(76) $$\begin{array}{cc} E^{\mathrm{Eq}} & :=\frac{\partial \rho {\overset{ˇ}{\psi }}^{\mathrm{pol},P}}{\partial P}=-T\frac{\partial \rho \overset{ˇ}{\eta }}{\partial P}\;. \end{array}$$

Then, we consider a change of variables by means of the Legendre transformation:

(77) $$\begin{array}{cc} \rho \psi^{\mathrm{pol}} & =\rho \psi^{\mathrm{pol},P}-E^{\mathrm{Eq}}\cdot P\;, \end{array}$$

such that we have the following representation and relations:

(78) $$\begin{array}{cccc} \rho \psi^{\mathrm{pol}} & =\rho {\overset{˘}{\psi }}^{\mathrm{pol}}(T,\;{\left(\rho_{\alpha }\right)}_{\alpha \in I},|E^{\mathrm{Eq}}\left|\right)\;, & \frac{\partial \rho {\overset{˘}{\psi }}^{\mathrm{pol}}}{\partial E^{\mathrm{Eq}}} & =-P\;. \end{array}$$

We introduce the scalar dielectric susceptibility as

(79) $$\begin{array}{cc} \chi & =-\frac{1}{\varepsilon_{0}\left|E^{\mathrm{Eq}}\right|}\frac{\partial }{\partial |E^{\mathrm{Eq}}|}\rho {\overset{˘}{\psi }}^{\mathrm{pol}}(T,{\left(\rho_{\alpha }\right)}_{\alpha \in I},|E^{\mathrm{Eq}}\left|\right)\;, \end{array}$$

such that, by (78), this implies, for the vector of polarisation, that

(80) $$\begin{array}{cc} P & =\chi (T,{\left(\rho_{\alpha }\right)}_{\alpha \in I},|E^{\mathrm{Eq}}\left|\right)\cdot \varepsilon_{0}E^{\mathrm{Eq}}\;. \end{array}$$

Considering the case of vanishing velocity, i.e., υ=0, the constitutive Equation (63) reduce to

(81) $$\begin{array}{cc} \tau^{P}\partial_{t}P & =+\left(\varepsilon_{0}E-\frac{1}{\chi }P\right)\;, \end{array}$$

where the relaxation constant $\tau^{P}>0$ is positive. From $\mathrm{div}\left(\varepsilon_{0}E+P\right)=n^{F}$, we conclude that, for the divergence of the constitutive Equation (81), it holds

(82) $$\begin{array}{cc} \tau^{P}\partial_{t}\mathrm{div}\left(P\right) & =n^{F}-\left(1+\frac{1}{\chi }\right)\mathrm{div}\left(P\right)\;. \end{array}$$

We conclude that, if $n^{F}=0$ in Equation (82), then div(P) vanishes for t→∞, which is compatible to stable polarisation relaxation. We remark that the stability of relaxation does not depend on the applied change of variables.

#### 5.3.2. Pressure, Surface Tension, and Stress Tensor

In the context of electrothermodynamics, the proper definition of pressure is not obvious. We define here pressure based on only these contributions, which are also present in the absence of electromagnetic fields. By the Gibbs–Duhem relation known from classical thermodynamics and its counterpart on the surface (cf. [9,10]), we define pressure and surface tension as

(83) $$\begin{array}{cccc} \hat{p} & =-\rho \hat{\psi }+\sum_{\alpha \in I^{\pm }}\rho_{\alpha }{\hat{\mu }}_{\alpha }\;, & \underset{s}{\gamma }\;\; & =\underset{s}{\rho }\;\;\underset{s}{\psi }\;\;-\sum_{\alpha \in I_{S}}\underset{s}{\rho }\;{\;}_{\alpha }\underset{s}{\mu }\;{\;}_{\alpha }\;, \end{array}$$

where the splitting of the free energy in (75) implies a similar splitting of the chemical potentials. The Legendre transformation (56) implies

(84a) $$\begin{array}{ccccc} \mu_{\alpha }\; & =\frac{\partial \rho \hat{\psi }}{\partial \rho_{\alpha }}+\frac{\partial \rho {\overset{˘}{\psi }}^{\mathrm{pol}}}{\partial \rho_{\alpha }} & \; & =-T\frac{\partial \rho \tilde{\eta }}{\partial \rho_{\alpha }}\;, & \end{array}$$

(84b) $$\begin{array}{cccc} \underset{s}{\mu }\;{\;}_{\alpha } & =\frac{\partial \underset{s}{\rho }\;\;\underset{s}{\hat{\psi }}\;\;}{\partial \underset{s}{\rho }\;{\;}_{\alpha }} & \; & =-\underset{s}{T}\;\;\frac{\partial \underset{s}{\rho }\;\;\underset{s}{\tilde{\eta }}\;\;}{\partial \underset{s}{\rho }\;{\;}_{\alpha }} \end{array}$$

With the above definitions of pressure and surface tension, we can rewrite the stress tensor in the volume in (65) and on the surface in (71) as

(85a) $$\begin{array}{cc} \Sigma -\sigma^{\mathrm{EM}}\; & =T-\left(\hat{p}-\rho {\overset{˘}{\psi }}^{\mathrm{pol}}+\sum_{\alpha \in I^{\pm }}\rho_{\alpha }\frac{\partial \rho {\overset{˘}{\psi }}^{\mathrm{pol}}}{\partial \rho_{\alpha }}\right)\;1+\frac{1}{2}\left(E⊗P+P⊗E\right)\;-P\cdot \left(E-E^{\mathrm{Eq}}\right)\;1\;, \end{array}$$

(85b) $$\begin{array}{cc} S^{\Gamma \Delta } & =\underset{s}{T}\;{\;}^{\Gamma \Delta }+\underset{s}{\gamma }\;\;\;g^{\Gamma \Delta }\;. \end{array}$$

Using the scalar susceptibility χ according to (79) and assuming polarisation relaxation equilibrium, i.e., $E^{\mathrm{Eq}}=E$, such that $P=\chi \varepsilon_{0}E$, according to (80), we infer with the Maxwell stress tensor according to (31) that

(86) $$\begin{array}{cc} \Sigma \; & =-\hat{p}\;1\;+T\;+\sigma^{\mathrm{EM}}\;+\chi \varepsilon_{0}E⊗E\;+\left(\rho {\overset{˘}{\psi }}^{\mathrm{pol}}-\sum_{\alpha \in I^{\pm }}\rho_{\alpha }\frac{\partial \rho {\overset{˘}{\psi }}^{\mathrm{pol}}}{\partial \rho_{\alpha }}\right)\;1\;. \end{array}$$

Due to the structural similarity to (83), one might refer to the last bracket in (86) as polarisation pressure. Nevertheless, we prefer to keep this term separate from the pressure contribution $\hat{p}$ to the stress tensor and relate the last terms in (83) to forces in the momentum balance. In the particular case of constant χ, we check $\rho {\overset{˘}{\psi }}^{\mathrm{pol}}=-\frac{1}{2}\chi \varepsilon_{0}{\left|E\right|}^{2}$ such that the stress tensor further simplifies to $\Sigma =-\hat{p}\;1\;+T+\left(1+\chi \right)\sigma^{\mathrm{EM}}$.

#### 5.3.3. Application to Momentum Balance

We again assumed $E^{\mathrm{Eq}}=E$ and $P=\chi \varepsilon_{0}E$. The divergence of the Maxwell stress tensor $\sigma^{\mathrm{EM}}$ equals in the quasi-electrostatic limit the Lorenz force $n^{e}E$, and it seems appropriate to treat also the divergence of the last two terms in (86) as forces in the momentum balance. Whenever χ is independent of the concentrations, i.e., independent of all $\rho_{\alpha }$ for α∈I, we apply $\nabla \rho {\overset{˘}{\psi }}^{\mathrm{pol}}=-\left(\nabla E^{\mathrm{Eq}}\right)\;P=-\chi \left(\nabla E\right)\;\varepsilon_{0}E$, to conclude, for the divergence of the stress tensor in the momentum balance,

(87) $$\begin{array}{cccc} \; & \underline{\mathrm{if}\;\chi \;\mathrm{is}\;\mathrm{independent}\;\mathrm{of}\;\mathrm{all}\;\rho_{\alpha }:} & \mathrm{div}(\Sigma )\; & =-\nabla \hat{p}+\mathrm{div}\left(T\right)\;+\underset{=n^{F}}{\underset{︸}{\mathrm{div}(\left(1+\chi \right)\varepsilon_{0}E)}}E\;. \end{array}$$

Due to its similarity, the term $n^{F}E$ is also referred to as the Lorentz force. On the other hand, if $\rho {\overset{˘}{\psi }}^{\mathrm{pol}}$ is a homogeneous function of degree one with respect to all $\rho_{\alpha }$ for α∈I, then χ is a homogeneous function as well, and the terms in the brackets in (86) cancel. We conclude

(88) $$\begin{array}{cccc} \; & \underline{\mathrm{if}\;\rho {\overset{˘}{\psi }}^{\mathrm{pol}}\;\mathrm{is}\;a\;\mathrm{homogeneous}\;\mathrm{function}\;\mathrm{of}\;\rho_{\alpha }:} & \mathrm{div}(\Sigma )\; & =-\nabla \hat{p}+\mathrm{div}\left(T\right)\;+n^{F}E+\chi \left(\nabla E\right)\varepsilon_{0}E\;. \end{array}$$

In addition to the Lorenz force, the last term in (88) is referred to as the Kelvin polarisation force. In the momentum balance on the surface, we have

(89) $$\begin{array}{cc} (\underset{s}{\sigma }\;{\;}^{i\Delta }{)}_{∥\Delta }\; & =\underset{s}{\gamma }\;{\;}_{∥\Delta }\;g^{\Gamma \Delta }\;\tau_{\Gamma }^{i}\;+2\underset{s}{\gamma }\;\;\;k_{M}\;\nu^{i}\;. \end{array}$$

## 6. Constitutive Equations for the Quasi-Magnetostatic Limit

The construction of constitutive relations for the quasi-magnetostatic limit follows largely the same lines as described in the previous section. The entropy principle is now based on constitutive functions of the entropy density in the form:

(90) $$\begin{array}{cccc} \rho \eta & =\rho \tilde{\eta }\left(\rho u,{\left(\rho_{\alpha }\right)}_{\alpha \in I^{\pm }},B\right)\;, & \underset{s}{\rho }\;\;\underset{s}{\eta }\;\; & =\underset{s}{\rho }\;\;\underset{s}{\tilde{\eta }}\;\;\left(\underset{s}{\rho }\;\;\underset{s}{u}\;\;,{\left(\underset{s}{\rho }\;{\;}_{\alpha }\right)}_{\alpha \in I_{S}}\right)\;. \end{array}$$

The (absolute) temperature $T,\underset{s}{T}\;\;$ and the chemical potentials $\mu_{\alpha },\underset{s}{\mu }\;{\;}_{\alpha }$ are defined in an analogous way as in (55), and we introduce the free energy in an analogous way as in (56). More specifically, we restrict the constitutive function for the free energy to the structure:

(91) $$\begin{array}{c} \rho \psi =\hat{\rho \psi }\left(T,{\left(n_{\alpha }\right)}_{\alpha \in I}\right)+\rho \psi^{\mathrm{mag}}(T,{\left(n_{\alpha }\right)}_{\alpha \in I},|B|)\;, \end{array}$$

such that the splitting of the free energy implies a similar splitting of the chemical potentials. As in (83), we define

(92) $$\begin{array}{cccc} \hat{p} & =-\rho \hat{\psi }+\sum_{\alpha \in I^{\pm }}\rho_{\alpha }{\hat{\mu }}_{\alpha }\;, & \underset{s}{\gamma }\;\; & =\underset{s}{\rho }\;\;\underset{s}{\psi }\;\;-\sum_{\alpha \in I_{S}}\underset{s}{\rho }\;{\;}_{\alpha }\underset{s}{\mu }\;{\;}_{\alpha }\;. \end{array}$$

### 6.1. Constitutive Relations for the Bulk

Taking the same entropy flux as above, the entropy production mostly looks the same as in (60), and we identify, again, five dissipative mechanism, where only polarisation is now replaced by magnetisation. We proceed in the same way as in Section 5 and apply the closure relations to each dissipative mechanism. For the reactions, there is no change to (62).

#### 6.1.1. Thermodiffusion

Compared to (61a), there is only a difference in the driving force of the migration term. We choose coefficients κ, $L_{\beta }$, and $M_{\alpha \beta }$ such that the coefficient matrix is symmetric and positive definite. We set

(93a) $$\begin{array}{cc} Q=\; & -\frac{\kappa }{T^{2}}\nabla T-\sum_{\beta \in I^{\pm }\setminus \left\{A_{0\pm }\right\}}L_{\beta }\left(\nabla \left(\frac{\mu_{\beta }}{T}-\frac{\mu_{0\pm }}{T}\right)-\frac{1}{T}\left(\frac{z_{\beta }e_{0}}{m_{\beta }}-\frac{z_{0\pm }e_{0}}{m_{0\pm }}\right)\left(\upsilon \times B\right)\right)\;, \end{array}$$

(93b) $$\begin{array}{cc} J_{\alpha }=\; & -\frac{L_{\alpha }}{T^{2}}\nabla T-\sum_{\beta \in I^{\pm }\setminus \left\{A_{0\pm }\right\}}M_{\alpha \beta }\left(\nabla \left(\frac{\mu_{\beta }}{T}-\frac{\mu_{0\pm }}{T}\right)-\frac{1}{T}\left(\frac{z_{\beta }e_{0}}{m_{\beta }}-\frac{z_{0\pm }e_{0}}{m_{0\pm }}\right)\left(\upsilon \times B\right)\right)\;. \end{array}$$

#### 6.1.2. Magnetisation

The entropy production due to magnetisation is

(94) $$\begin{array}{cc} \xi_{M}\; & =+{\left(\frac{\partial \rho \tilde{\eta }}{\partial B}-\frac{1}{T}M\right)\cdot \left(\partial_{t}B+\left(\upsilon \cdot \nabla \right)B-\frac{1}{2}\left(\nabla \upsilon -\nabla \upsilon^{T}\right)B\right)}_{}\;. \end{array}$$

We choose a linear closure relation with the relaxation time $\tau^{M}>0$ of magnetisation to obtain

(95) $$\begin{array}{cc} \tau^{M}\frac{1}{\mu_{0}}\left(\partial_{t}B+\left(\upsilon \cdot \nabla \right)B-\frac{1}{2}\left(\nabla \upsilon -\nabla \upsilon^{T}\right)B\right)\; & =T\frac{\partial \rho \tilde{\eta }}{\partial B}-M\;. \end{array}$$

We introduce the conjugate variable $M^{\mathrm{Eq}}$ and the scalar magnetic susceptibility $\chi_{M}$ as

(96) $$\begin{array}{cccc} M^{\mathrm{Eq}} & :=-\frac{\partial \rho \psi }{\partial B}=T\frac{\partial \rho \overset{ˇ}{\eta }}{\partial B}\;, & \frac{\chi_{M}}{1+\chi_{M}} & =-\frac{\mu_{0}}{\left|B\right|}\frac{\partial }{\partial |B|}\rho \psi^{\mathrm{mag}}(T,{\left(n_{\alpha }\right)}_{\alpha \in I},|B|)\;, \end{array}$$

where $\chi_{M}=\chi_{M}(T,{\left(n_{\alpha }\right)}_{\alpha \in I},|B|)$, and we have

(97) $$\begin{array}{cc} M^{\mathrm{Eq}}\; & =\frac{\chi_{M}}{1+\chi_{M}}\cdot \frac{1}{\mu_{0}}B\;. \end{array}$$

In magnetisation, equilibrium (94) implies $M^{\mathrm{Eq}}=M$. When we consider the case of vanishing velocity, i.e., υ=0, the constitutive Equation (95) simplifies to

(98) $$\begin{array}{cc} \tau^{M}\frac{1}{\mu_{0}}\partial_{t}B & =-\left(-\frac{\chi_{M}}{1+\chi_{M}}\frac{1}{\mu_{0}}B+M\right)\;, \end{array}$$

From $\mathrm{curl}\left(\frac{1}{\mu_{0}}B-M\right)=J^{F}$, we conclude for the curl of (98) that

(99) $$\begin{array}{cc} \tau^{M}\partial_{t}\mathrm{curl}\left(B\right)\; & =J^{F}-\left(-\frac{\chi_{M}}{1+\chi_{M}}+1\right)\mathrm{curl}\left(B\right)\;. \end{array}$$

We conclude the stable magnetisation relaxation for $J^{F}=0$ and $\chi_{M}>-1$, i.e., curl(B) vanishes for t→∞. While $\chi_{M}>0$ represents the paramagnetic material behaviour, $\chi_{M}<0$ characterises diamagnetic materials. The limit $\chi_{M}=-1$ represents superconducting materials.

#### 6.1.3. Viscosity

Taking for the viscous stress tensor the same approach as in (64), i.e., $T=\eta_{b}\mathrm{div}\left(\upsilon \right)1+\eta_{s}\left(\nabla \upsilon +{\left(\nabla \upsilon \right)}^{T}\right)$, yields, for the symmetric total stress tensor Σ in the quasi-magnetostatic limit, the constitutive equation:

(100) $$\begin{array}{cc} \Sigma -\sigma^{\mathrm{EM}}\; & =T+\left(\rho u-T\rho \tilde{\eta }+M\cdot B-\sum_{\alpha \in I^{\pm }}\rho_{\alpha }\mu_{\alpha }\right)\;1-\frac{1}{2}\left(M⊗B+B⊗M\right)\;. \end{array}$$

With the definition of pressure according to (92), the free energy as in (91) with the scalar magnetic susceptibility according to (96), and assuming magnetisation relaxation equilibrium, i.e., $M^{\mathrm{Eq}}=-M$, we obtain

(101) $$\begin{array}{cc} \Sigma \; & =-\hat{p}\;1\;+T\;+\sigma^{\mathrm{EM}}\;-\frac{\chi_{M}}{1+\chi_{M}}\frac{1}{\mu_{0}}B⊗B\;+\left(\rho \psi^{\mathrm{mag}}-\sum_{\alpha \in I^{\pm }}\rho_{\alpha }\frac{\partial \rho \psi^{\mathrm{mag}}}{\partial \rho_{\alpha }}+\frac{\chi_{M}}{1+\chi_{M}}\frac{1}{\mu_{0}}{\left|B\right|}^{2}\right)\;1\;, \end{array}$$

where, in the case of constant $\chi_{M}$, we have $\rho \psi^{\mathrm{mag}}=-\frac{\chi_{M}}{1+\chi_{M}}\frac{1}{2\mu_{0}}{\left|B\right|}^{2}$ and the stress tensor further simplifies to $\Sigma =-\hat{p}\;1\;+T\;+\frac{1}{1+\chi_{M}}\sigma^{\mathrm{EM}}$.

The divergence of the Maxwell stress tensor $\sigma^{\mathrm{EM}}$ equals in the quasi-magnetostatic limit the Lorenz force $(n^{e}\upsilon +J^{e})\times B$, and it seems appropriate to treat also the divergence of the last two terms in (101) as forces in the momentum balance.

### 6.2. Constitutive Relations for the Surface

We use the definitions of $\underset{s}{T}\;\;$ and $\underset{s}{D}\;\;$ as in (66) to derive the surface entropy production in an analogously way as before. For the surface reactions, there is no change to (69), and for the viscous stress tensor on the surface, there is no change to (70).

#### 6.2.1. Thermodiffusion

With the same choice of the symmetric and positive-definite coefficient matrix, we set, as in (68) for $\alpha \in I_{S}\setminus \left\{A_{0}\right\}$,

(102a) $$\begin{array}{cc} \underset{s}{q}\;{\;}_{\tau }^{\Delta }=\; & -\frac{\underset{s}{\kappa }\;\;}{\underset{s}{T}\;{\;}^{2}}g^{\Delta \Gamma }\underset{s}{T}\;{\;}_{∥\Gamma }\;-\sum_{\beta \in I_{S}\setminus \left\{A_{0}\right\}}\underset{s}{L}\;{\;}_{\beta }\left[g^{\Delta \Gamma }{\left(\frac{\underset{s}{\mu }\;{\;}_{\beta }}{\underset{s}{T}\;\;}-\frac{\underset{s}{\mu }\;{\;}_{0}}{\underset{s}{T}\;\;}\right)}_{∥\Gamma }-\frac{1}{\underset{s}{T}\;\;}\left(\frac{z_{\beta }e_{0}}{m_{\beta }}-\frac{z_{0}e_{0}}{m_{0}}\right){\left(\underset{s}{\upsilon }\;\;\times \bar{B}\right)}_{\tau }^{\Delta }\right], \end{array}$$

(102b) $$\begin{array}{cc} \underset{s}{J}\;{\;}_{\alpha ,\tau }^{\Delta }=\; & -\frac{\underset{s}{L}\;{\;}_{\alpha }}{\underset{s}{T}\;{\;}^{2}}g^{\Delta \Gamma }\underset{s}{T}\;{\;}_{∥\Gamma }\;-\sum_{\beta \in I_{S}\setminus \left\{A_{0}\right\}}\underset{s}{M}\;{\;}_{\alpha \beta }\left[g^{\Delta \Gamma }{\left(\frac{\underset{s}{\mu }\;{\;}_{\beta }}{\underset{s}{T}\;\;}-\frac{\underset{s}{\mu }\;{\;}_{0}}{\underset{s}{T}\;\;}\right)}_{∥\Gamma }-\frac{1}{\underset{s}{T}\;\;}\left(\frac{z_{\beta }e_{0}}{m_{\beta }}-\frac{z_{0}e_{0}}{m_{0}}\right){\left(\underset{s}{\upsilon }\;\;\times \bar{B}\right)}_{\tau }^{\Delta }\right]\;. \end{array}$$

#### 6.2.2. Stress Coming from the Volume

We choose coefficients $\underset{s}{\lambda }\;{\;}^{\pm }$ and $\underset{s}{\eta }\;{\;}_{\Delta }^{\pm }>0$, for Δ=1,2, and obtain, from the linear closure,

(103a) $$\begin{array}{cc} \; & {\left(\nu \cdot \left(T+\frac{1}{2}\left(M⊗B-B⊗M\right)\right)\tau_{\Gamma }\;g^{\Gamma \Delta }\right)}^{\pm }=\pm \underset{s}{\eta }\;{\;}_{\Delta }^{\pm }\;{\left(\upsilon_{\tau }^{\Delta }-\underset{s}{\upsilon }\;{\;}_{\tau }^{\Delta }\right)}^{\pm }\;,\\ \; & {\left(\nu \cdot \left(T+\frac{1}{2}\left(M⊗B-B⊗M\right)\right)\nu -\left(\frac{1}{2}\rho {\left|\underset{s}{\upsilon }\;\;-\upsilon \right|}^{2}+\underset{s}{T}\;\;\rho \left(\frac{\mu_{0\pm }}{T}-\frac{\underset{s}{\mu }\;{\;}_{0\pm }}{\underset{s}{T}\;\;}\right)\right)\right)}^{\pm } \end{array}$$

(103b) $$\begin{array}{cc} \; & \;=\pm \underset{s}{\lambda }\;{\;}^{\pm }\rho^{\pm }\;{\left(\rho \;(\upsilon_{\nu }-\underset{s}{\upsilon }\;{\;}_{\nu })\right)}^{\pm }\;. \end{array}$$

## 7. Summary and Discussion

By asymptotic considerations, we motivated the formulation of two distinct Galilean limit systems of the Maxwell equations. Both Galilean limits are well known for volume domains and, here, were transferred to surfaces. Due to their transformation properties, each one of these limit systems can easily be coupled in a consistent manner to standard non-relativistic balance equations of matter that are well suited for most engineering applications. Then, constitutive equations to close the coupled system can be obtained from an entropy principle.

### 7.1. Comparison to the Results of [8]

Considering first the quasi-electrostatic limit, the same results as in Section 5, in principle, can also be been obtained by [8], where the full Maxwell equations are employed, and the quasi-electrostatic limit, then, can be taken after the application of the entropy principle. However, this procedure is less straightforward, because in [8], the inner energy density of the coupled system is redefined in order to obtain the entropy production related to magnetisation as a binary product satisfying a Galilean symmetry principle. Such a modification of the inner energy as ρu+M·B seems admissible because, in coupled electrothermodynamics, it is not a priori evident what is the correct definition of the inner energy. The non-dimensionalisation of Section 3.3 reveals that this modification made to the inner energy is asymptotically small in the quasi-electrostatic limit and can, therefore, be neglected.

An alternative application of the entropy principle based on E as the independent variable was discussed in [8]. It relies on a different definition of the inner energy as ρu−E·P, and therefore, it is fundamentally different from the change of variables employed here in Section 5.3. The term E·P is not asymptotically small compared to ρu in the quasi-electrostatic limit and, thus, implies a non-negligible difference in the definition of the temperature. Moreover, the application of a linear closure relation then yields an unstable relaxation of polarisation. In contrast, the change of variables applied here in Section 5.3 and in the same way also in [7], changing the variables from P to $E^{\mathrm{Eq}}$, does not alter the stability of polarisation relaxation, and in equilibrium, we have $E^{\mathrm{Eq}}=E$.

In addition, the entropy principle in [8] also covers magnetisation. We omitted magnetisation here in Section 5, because, in the quasi-electrostatic limit, the corresponding contributions to the entropy production are in the asymptotic order of the terms that we neglected in the balance equations. Without a constitutive equation relating B and M, we are in general not able to evaluate the electric current $j^{e}$, although, in many experiments, this is the observable quantity of pivotal importance. Therefore, either an additional constitutive law has to be obtained from a different source, or we have to apply an additional asymptotic limit such as the thin double-layer limit, where the contribution to $j^{e}$ due to M vanishes. The relaxation of magnetisation was not discussed in [8]. We note that an analogous approach as performed here in Section 6, but applied to the main approach of [8] based on the variables (P,M), would imply the blow up of curl(M) for a diamagnetic material.

In the quasi-magnetostatic limit, we assumed in Section 6 the entropy density to depend on the independent variable E. This is different than the main approach of [8], but compatible with the alternative approach discussed there and with [10]. Within the quasi-magnetostatic limit, the definition of the inner energy as ρu−E·P is a negligible modification, whereas M·B is not asymptotically small. Accordingly, we observed in Section 6 the stable relaxation of magnetisation, whereas the approach based on the variable M would lead to unstable relaxation for a diamagnetic material.

Each of the modifications of the inner energy in [8] implies according changes to the stress tensor when it is expressed in terms of free energy. Non-constant susceptibility requires careful treatment of the momentum balance; cf. [7].

### 7.2. Conclusions for Coupling with General Maxwell Equations

Coupling the full Maxwell equations to the non-relativistic balance equations of matter appears to be a more general approach than the one presented here. For this general case, two alternative formulations of the entropy principle were analysed in [8], one based on the variables (P,M) and the other one based on the variables (E,B). While the (P,M)-variant allows reaching the quasi-electrostatic Galilean limit where only P remains as a variable, the (E,B)-variant allows reaching the quasi-magnetostatic Galilean limit with only B as the remaining variable. Neither of the two variants is capable of covering both limit cases equally well, as for the (P,M)-variant, magnetisation relaxation is unstable for a diamagnetic material, whereas, for the (E,B)-variant, polarisation relaxation is unstable. However, this should not be considered a source of inconsistency for the limit cases, as the two Galilean limits do not have any relevant overlap. We remark that building the entropy principle on (P,B) as independent variables does not provide a remedy for the general case, since P and B do not form an antisymmetric four-tensor in the four-dimensional formulation of the Maxwell equations. Therefore, we conclude that, *if* the electric and magnetic effects both are relevant in the application, *then* one is well advised to consider, in addition to the general Maxwell equations, also a relativistic description of the mixture of matter. This leaves as an open question how to find appropriate models for, e.g., liquid metal batteries or magneto-hydrodynamic forces in the electrolyte transport of the Hall–Heroult process.

### 7.3. Electrochemical Model in Polarisation Equilibrium

We summarise here the complete system of the model equations for electrochemical applications under the simplifying assumption of fast polarisation relaxation. An important feature of electrochemical systems is the formation of double-layers at the contact of different materials. The double-layer is characterised by a typical width in the range of nanometres, wherein the electric potential may vary in the order of one Volt. The magnetic field strength is assumed to be below a guideline value for electromagnetic fields in electrical household appliances. These reference values:

(104) $$\begin{array}{cccccc} B^{\mathrm{ref}} & =10^{-3}\frac{\;V\;s}{m^{2}}\;, & E^{\mathrm{ref}} & =10^{9}\frac{\;V}{m}\;, & c_{0} & \approx 3\cdot 10^{8}\frac{m}{\;s}\;, \end{array}$$

imply, for the dimensionless quantity,

(105) $$\begin{array}{cc} \beta^{2} & \approx 3\cdot 10^{-4}\ll 1\;. \end{array}$$

The smallness of $\beta^{2}$ suggests the use of the quasi-electrostatic limit, where (29b) implies the existence of an *electrostatic potential* φ, such that

(106) $$\begin{array}{cccc} E & =-\nabla \varphi \;, & \left[\;[\varphi ]\;\right] & =const.\;\mathrm{on}\;S\;, \end{array}$$

where the constant is frequently chosen as zero.

For each volume domain $\Omega^{\pm }$, we use index sets $I^{\pm }$ for the constituents of the mixture. The constitutive equations in the volume are built on the free energy density of the structure:

(107) $$\begin{array}{cc} \rho \psi & =\hat{\rho \psi }\left(T,{\left(\rho_{\alpha }\right)}_{\alpha \in I}\right)+\rho \psi^{\mathrm{pol}}(T,{\left(\rho_{\alpha }\right)}_{\alpha \in I},|\nabla \varphi |)\;, \end{array}$$

The chemical potentials for $\alpha \in I^{\pm }$, pressure, and the inner energy are given in terms of the free energy as

(108) $$\begin{array}{cccccc} \mu_{\alpha } & =\frac{\partial }{\partial \rho_{\alpha }}\rho \psi \;, & p & =-\rho \hat{\psi }+\sum_{\alpha \in I^{\pm }}\rho_{\alpha }{\hat{\mu }}_{\alpha }\;, & \rho u & =\left(T^{2}\frac{\partial }{\partial T}\frac{\rho \psi }{T}\right)\;. \end{array}$$

The dielectric susceptibility is defined as

(109) $$\begin{array}{cc} \chi & =-\frac{1}{\varepsilon_{0}\left|\nabla \varphi \right|}\frac{\partial }{\partial |\nabla \varphi |}\rho \psi^{\mathrm{pol}}(T,{\left(\rho_{\alpha }\right)}_{\alpha \in I},|\nabla \varphi |)\;. \end{array}$$

Since polarisation relaxation in liquid electrolytes is typically fast with a time constant in the range of $10^{-8}\;s$ (cf. [20]), we can assume that polarisation relaxation is in quasi-equilibrium, i.e., $P=-\chi \varepsilon_{0}\nabla \varphi$. In the following, we distinguish two cases:

(110) $$\begin{array}{c} \chi \;\mathrm{is}\;\left\{\begin{array}{cc} \;\mathrm{independent}\;\mathrm{of}\;\rho_{\alpha }\;, & \mathrm{indicated}\;\mathrm{as}\;\;(*)\;,\\ \;\mathrm{homogeneous}\;\mathrm{of}\;\mathrm{degree}\;\mathrm{one}\;\mathrm{in}\;\rho_{\alpha }\;, & \mathrm{indicated}\;\mathrm{as}\;\;(**)\;. \end{array}\right. \end{array}$$

In each volume domain, we chose a constituent $A_{0,\pm }\in I^{\pm }$. Then, the balance equations are

(111a) $$\begin{array}{cc} -\mathrm{div}(\left(1+\chi \right)\varepsilon_{0}\nabla \varphi ) & =n^{F}\;, \end{array}$$

(111b) $$\begin{array}{cc} \partial_{t}\rho_{\alpha }+\mathrm{div}\left(\rho_{\alpha }\upsilon +J_{\alpha }\right)\; & =m_{\alpha }\sum_{k}\gamma_{\alpha }^{k}R^{k}\;\;\mathrm{for}\;\alpha \in I^{\pm }\setminus \left\{A_{0\pm }\right\}\;, \end{array}$$

(111c) $$\begin{array}{ccc} \partial_{t}\rho +\mathrm{div}\left(\rho \upsilon \right)\; & =0\;, & \end{array}$$

(111d) $$\begin{array}{cc} \partial_{t}\left(\rho \upsilon \right)+\mathrm{div}\left(\rho \upsilon ⊗\upsilon -T\right)+\nabla p\; & =\rho f-n^{F}\nabla \varphi +\left\{\begin{array}{cc} 0\;, & \;(*)\;,\\ \left(D^{2}\varphi \right)\;\chi \varepsilon_{0}\nabla \varphi \;, & \;(**)\;, \end{array}\right.\\ \partial_{t}\left(\rho u\right)+\mathrm{div}\left(\rho u\;\upsilon +Q\right)\; & =-J^{F}\cdot \nabla \varphi \;+T:\nabla \upsilon \;-p\;\mathrm{div}\left(\upsilon \right) \end{array}$$

(111e) $$\begin{array}{cc} \; & +\left(\partial_{t}\left(\chi \varepsilon_{0}\nabla \varphi \right)+\left(\upsilon \cdot \nabla \right)\left(\chi \varepsilon_{0}\nabla \varphi \right)\right)\cdot \nabla \varphi \\ \; & +\mathrm{div}\left(\upsilon \right)\left\{\begin{array}{cc} \chi \varepsilon_{0}{\left|\nabla \varphi \right|}^{2}-\rho \psi^{\mathrm{pol}}\;, & \;(*)\;,\\ \chi \varepsilon_{0}{\left|\nabla \varphi \right|}^{2}\;, & \;(**)\;, \end{array}\right. \end{array}$$

where $D^{2}\varphi$ denotes the Hessian containing the second derivatives. The constitutive equations in the volume are

(112a) $$\begin{array}{cc} Q & =\;-\frac{\kappa }{T^{2}}\nabla T\;-\;\;\sum_{\beta \in I^{\pm }\setminus \left\{A_{0\pm }\right\}}L_{\beta }\left(\nabla \left(\frac{\mu_{\beta }}{T}-\frac{\mu_{0\pm }}{T}\right)+\frac{e_{0}}{T}\left(\frac{z_{\beta }}{m_{\beta }}-\frac{z_{0\pm }}{m_{0\pm }}\right)\nabla \varphi \right)\;, \end{array}$$

(112b) $$\begin{array}{cc} J_{\alpha } & =-\frac{L_{\alpha }}{T^{2}}\nabla T\;-\;\;\sum_{\beta \in I^{\pm }\setminus \left\{A_{0\pm }\right\}}M_{\alpha \beta }\left(\nabla \left(\frac{\mu_{\beta }}{T}-\frac{\mu_{0\pm }}{T}\right)+\frac{e_{0}}{T}\left(\frac{z_{\beta }}{m_{\beta }}-\frac{z_{0\pm }}{m_{0\pm }}\right)\nabla \varphi \right)\;, \end{array}$$

(112c) $$\begin{array}{cc} T & =\eta_{b}\;\mathrm{div}\left(\upsilon \right)1\;+\eta_{s}\cdot \left(\nabla \upsilon +{\left(\nabla \upsilon \right)}^{T}\right)\;, \end{array}$$

(112d) $$\begin{array}{cc} R^{k} & =R_{0}^{k}\cdot \left(1-\exp\left(\frac{A^{k}}{k_{B}T}\sum_{\alpha \in I^{\pm }}\gamma_{\alpha }^{k}\;m_{\alpha }\mu_{\alpha }\right)\right)\;. \end{array}$$

On the surface with the index set $I_{S}$ for the constituents, the free energy density does not depend on the electric field. The chemical potentials for $\alpha \in I_{S}$, surface tension, and the inner energy are given in terms of the free energy as

(113) $$\begin{array}{c} \underset{s}{\rho }\;\;\underset{s}{\psi }\;\;=\underset{s}{\rho }\;\;\underset{s}{\hat{\psi }}\;\;\left(T,{\left(\underset{s}{\rho }\;{\;}_{\alpha }\right)}_{\alpha \in I_{S}}\right)\;,\;\underset{s}{\mu }\;{\;}_{\alpha }=\frac{\partial }{\partial \underset{s}{\rho }\;{\;}_{\alpha }}\underset{s}{\rho }\;\;\underset{s}{\hat{\psi }}\;\;\;,\;\underset{s}{\gamma }\;\;=\underset{s}{\rho }\;\;\underset{s}{\psi }\;\;-\sum_{\alpha \in I_{S}}\underset{s}{\rho }\;{\;}_{\alpha }\underset{s}{\mu }\;{\;}_{\alpha }\;,\;\underset{s}{\rho }\;\;\underset{s}{u}\;\;=\left(\underset{s}{T}\;{\;}^{2}\frac{\partial }{\partial \underset{s}{T}\;\;}\frac{\underset{s}{\rho }\;\;\underset{s}{\hat{\psi }}\;\;}{\underset{s}{T}\;\;}\right)\;. \end{array}$$

We choose a constituent $A_{0}\in I_{S}$ and let $\alpha \in I_{S}\setminus \left\{A_{0}\right\}$. Then, the surface balance equations are

(114a) $$\begin{array}{ccc} -\varepsilon_{0}\left[\;\left[\left(1+\chi \right)\nabla \varphi \cdot \nu \right]\;\right] & =\underset{s}{n}\;{\;}^{F}\;, & \\ \partial_{t,\nu }\underset{s}{\rho }\;{\;}_{\alpha }+{\left(\underset{s}{\rho }\;{\;}_{\alpha }\underset{s}{\upsilon }\;{\;}_{\tau }^{\Delta }+\underset{s}{J}\;{\;}_{\alpha ,\tau }^{\Delta }\right)}_{∥\Delta }\; & \end{array}$$

(114b) $$\begin{array}{cc} -2k_{M}\underset{s}{\upsilon }\;{\;}_{\nu }\;\underset{s}{\rho }\;{\;}_{\alpha }\; & =-\left[\;\left[\rho_{\alpha }\left(\upsilon_{\nu }-\underset{s}{\upsilon }\;{\;}_{\nu }\right)+J_{\alpha }\cdot \nu \right]\;\right]+m_{\alpha }\sum_{\ell }\underset{s}{\gamma }\;{\;}_{\alpha }^{\ell }\underset{s}{R}\;{\;}^{\ell }\;, \end{array}$$

(114c) $$\begin{array}{ccc} \partial_{t,\nu }\underset{s}{\rho }\;\;+{\left(\underset{s}{\rho }\;\;\underset{s}{\upsilon }\;{\;}_{\tau }^{\Delta }\right)}_{∥\Delta }-2k_{M}\underset{s}{\upsilon }\;{\;}_{\nu }\;\underset{s}{\rho }\;\;\; & =-[\;\left[\rho \left(\upsilon_{\nu }-\underset{s}{\upsilon }\;{\;}_{\nu }\right)\right]\;]\;, & \\ \partial_{t,\nu }\left(\underset{s}{\rho }\;\;\underset{s}{\upsilon }\;{\;}^{i}\right)+{\left(\underset{s}{\rho }\;\;\underset{s}{\upsilon }\;{\;}^{i}\underset{s}{\upsilon }\;{\;}_{\tau }^{\Delta }-\underset{s}{T}\;{\;}^{\Gamma \Delta }\tau_{\Gamma }^{i}\right)}_{∥\Delta }\; & \end{array}$$

$$\begin{array}{cc} -2k_{M}\underset{s}{\upsilon }\;{\;}_{\nu }\;\underset{s}{\rho }\;\;\underset{s}{\upsilon }\;{\;}^{i}\; & =-[\;\left[\rho \upsilon^{i}\left(\upsilon_{\nu }-\underset{s}{\upsilon }\;{\;}_{\nu }\right)+p\nu^{i}-\left(T+\left(1+\chi \right)\sigma^{\mathrm{EM}}\right)\cdot \nu \right]\;] \end{array}$$

(114d) $$\begin{array}{ccc} \; & +2k_{M}\underset{s}{\gamma }\;\;\nu^{i}+\underset{s}{\gamma }\;{\;}_{∥\Delta }\;g^{\Gamma \Delta }\tau_{\Gamma }^{i}+\underset{s}{\rho }\;\;\underset{s}{f}\;{\;}^{i} & \\ \; & +[\;[\frac{1}{2}\chi \varepsilon_{0}{\left|\nabla \varphi \right|}^{2}\nu^{i}]\;]+\left\{\begin{array}{cc} {}[\;\left[\rho \psi^{\mathrm{pol}}\nu^{i}\right]\;]\;, & \;(*)\;,\\ 0\;, & \;(**)\;, \end{array}\right.\\ \partial_{t,\nu }\left(\underset{s}{\rho }\;\;\underset{s}{u}\;\;\right)+{\left(\underset{s}{\rho }\;\;\underset{s}{u}\;\;\;\underset{s}{\upsilon }\;{\;}_{\tau }^{\Delta }+\underset{s}{q}\;{\;}^{\Delta }\right)}_{∥\Delta }\; & \end{array}$$

(114e) $$\begin{array}{cc} -2k_{M}\underset{s}{\upsilon }\;{\;}_{\nu }\;\underset{s}{\rho }\;\;\underset{s}{u}\;\;\; & =\;-\underset{s}{J}\;{\;}^{F}\cdot \bar{\nabla \varphi }+\left(\underset{s}{T}\;{\;}^{\Gamma \Delta }+\underset{s}{\gamma }\;\;\;g^{\Gamma \Delta }\right)\tau_{\Gamma }^{i}\underset{s}{\upsilon }\;{\;}_{∥\Delta }^{i}\\ \; & -\left[\;\;\left[\left(\rho u+\frac{1}{2}\rho {\left|\upsilon -\underset{s}{\upsilon }\;\;\right|}^{2}\right)\;\left(\upsilon_{\nu }-\underset{s}{\upsilon }\;{\;}_{\nu }\right)+Q\cdot \nu \right]\;\;\right]\\ \; & +\left[\;\left[\left(\upsilon -\underset{s}{\upsilon }\;\;\right)\cdot \left(-p1+T\right)\cdot \nu \right]\;\right]\;.+\left\{\begin{array}{cc} {}[\;\left[{\left(\upsilon -\underset{s}{\upsilon }\;\;\right)}_{\nu }\;\rho \psi^{\mathrm{pol}}\right]\;]\;, & \;(*)\;,\\ 0\;, & \;(**)\;. \end{array}\right. \end{array}$$

The constitutive equations are, with $\alpha \in I^{\pm }\setminus \left\{A_{0\pm }\right\}$,

(115a) $$\begin{array}{cc} \underset{s}{q}\;{\;}_{\tau }^{\Delta } & =-\frac{\underset{s}{\kappa }\;\;}{\underset{s}{T}\;{\;}^{2}}g^{\Delta \Gamma }\underset{s}{T}\;{\;}_{∥\Gamma }\;-\;\;\sum_{\beta \in I_{S}\setminus \left\{A_{0}\right\}}\underset{s}{L}\;{\;}_{\beta }\left[g^{\Delta \Gamma }{\left(\frac{\underset{s}{\mu }\;{\;}_{\beta }}{\underset{s}{T}\;\;}-\frac{\underset{s}{\mu }\;{\;}_{0}}{\underset{s}{T}\;\;}\right)}_{∥\Gamma }+\frac{e_{0}}{\underset{s}{T}\;\;}\left(\frac{z_{\beta }}{m_{\beta }}-\frac{z_{0}}{m_{0}}\right){\bar{\nabla \varphi }}_{\tau }^{\Delta }\right], \end{array}$$

(115b) $$\begin{array}{cc} \underset{s}{J}\;{\;}_{\alpha ,\tau }^{\Delta } & =-\frac{\underset{s}{L}\;{\;}_{\alpha }}{\underset{s}{T}\;{\;}^{2}}g^{\Delta \Gamma }\underset{s}{T}\;{\;}_{∥\Gamma }\;-\;\;\sum_{\beta \in I_{S}\setminus \left\{A_{0}\right\}}\underset{s}{M}\;{\;}_{\alpha \beta }\left[g^{\Delta \Gamma }{\left(\frac{\underset{s}{\mu }\;{\;}_{\beta }}{\underset{s}{T}\;\;}-\frac{\underset{s}{\mu }\;{\;}_{0}}{\underset{s}{T}\;\;}\right)}_{∥\Gamma }+\frac{e_{0}}{\underset{s}{T}\;\;}\left(\frac{z_{\beta }}{m_{\beta }}-\frac{z_{0}}{m_{0}}\right){\bar{\nabla \varphi }}_{\tau }^{\Delta }\right]\;, \end{array}$$

(115c) $$\begin{array}{cc} \underset{s}{T}\;{\;}^{\Delta \Gamma } & =\underset{s}{\eta }\;{\;}_{b}\;\underset{s}{D}\;{\;}_{\Lambda \Sigma }\;g^{\Lambda \Sigma }\;g^{\Delta \Gamma }+2\underset{s}{\eta }\;{\;}_{s}\;g^{\Lambda \Delta }\underset{s}{D}\;{\;}_{\Lambda \Sigma }\;g^{\Sigma \Gamma }\;, \end{array}$$

(115d) $$\begin{array}{cc} \; & \mathrm{with}\;\underset{s}{D}\;{\;}_{\Delta \Gamma }=\frac{1}{2}\left(g_{\Gamma \Lambda }\;\underset{s}{\upsilon }\;{\;}_{\tau ∥\Delta }^{\Lambda }+g_{\Delta \Lambda }\;\underset{s}{\upsilon }\;{\;}_{\tau ∥\Gamma }^{\Lambda }\right)-b_{\Gamma \Delta }\;\underset{s}{\upsilon }\;{\;}_{\nu }\;, \end{array}$$

(115e) $$\begin{array}{cc} \underset{s}{R}\;{\;}^{\ell } & =\underset{s}{R}\;{\;}_{0}^{\ell }\cdot \left(\exp\left(-\frac{\beta^{\ell }}{k_{B}\underset{s}{T}\;\;}\underset{s}{D}\;{\;}^{\ell }\right)-\exp\left(\frac{1-\beta^{\ell }}{k_{B}\underset{s}{T}\;\;}\underset{s}{D}\;{\;}^{\ell }\right)\right)\;, \end{array}$$

(115f) $$\begin{array}{cc} \; & \mathrm{with}\;\underset{s}{D}\;{\;}^{\ell }=\underset{s}{A}\;{\;}^{\ell }\sum_{\alpha \in I_{S}}\underset{s}{\gamma }\;{\;}_{\alpha }^{\ell }\;m_{\alpha }\underset{s}{\mu }\;{\;}_{\alpha }\;. \end{array}$$

Moreover, there are the boundary conditions:

(116a) $$\begin{array}{cc} {\left(J_{\alpha ,\nu }+\rho_{\alpha }\left(\upsilon_{\nu }-\underset{s}{\upsilon }\;{\;}_{\nu }\right)\right)}^{\pm }\; & =\mp \underset{s}{M}\;{\;}_{\alpha }^{\pm }\left(\exp\left(\frac{\beta_{\alpha }^{\pm }}{k_{B}T}D_{\alpha }^{\pm }\right)-\exp\left(\frac{\beta_{\alpha }^{\pm }-1}{k_{B}T}D_{\alpha }^{\pm }\right)\right)\;, \end{array}$$

(116b) $$\begin{array}{ccc} \; & \mathrm{with}\;D_{\alpha }^{\pm }={\left(\mu_{\alpha }-\mu_{0\pm }-\frac{T}{\underset{s}{T}\;\;}\left(\underset{s}{\mu }\;{\;}_{\alpha }-\underset{s}{\mu }\;{\;}_{0\pm }\right)\right)}^{\pm }\;, & \end{array}$$

(116c) $$\begin{array}{cc} \pm \underset{s}{\eta }\;{\;}_{\Delta }^{\pm }\;{\left(\upsilon_{\tau }^{\Delta }-\underset{s}{\upsilon }\;{\;}_{\tau }^{\Delta }\right)}^{\pm }\; & ={\left(g^{\Gamma \Delta }\tau_{\Gamma }\cdot T\nu \right)}^{\pm }\;, \end{array}$$

(116d) $$\begin{array}{cc} \pm \underset{s}{\lambda }\;{\;}^{\pm }\rho^{\pm }\;{\left(\rho \;(\upsilon_{\nu }-\underset{s}{\upsilon }\;{\;}_{\nu })\right)}^{\pm }\; & ={\left(\nu \cdot T\nu -\frac{1}{2}\rho {\left|\underset{s}{\upsilon }\;\;-\upsilon \right|}^{2}-\underset{s}{T}\;\;\rho \;\left(\frac{\mu_{0\pm }}{T}-\frac{\underset{s}{\mu }\;{\;}_{0\pm }}{\underset{s}{T}\;\;}\right)\right)}^{\pm }\;, \end{array}$$

(116e) $$\begin{array}{cc} \pm \underset{s}{\kappa }\;{\;}^{\pm }\;{\left(\frac{1}{T}-\frac{1}{\underset{s}{T}\;\;}\right)}^{\pm }\; & ={\left(Q_{\nu }+\left(\rho u+p\right)\;\left(\upsilon_{\nu }-\underset{s}{\upsilon }\;{\;}_{\nu }\right)\right)}^{\pm }\\ \; & -\left\{\begin{array}{cc} {\left(\rho \psi^{\mathrm{pol}}\left(\upsilon_{\nu }-\underset{s}{\upsilon }\;{\;}_{\nu }\right)\right)}^{\pm }\;, & \;(*)\;,\\ 0\;, & \;(**)\;. \end{array}\right. \end{array}$$

Finally, we remark that the evaluation of the electric current, in general, also requires knowledge of the magnetic field, although the magnetic field does not have any influence on the solution of the above system. In order to compute B for a given solution of the above model equations, we assume that magnetisation relaxation is fast and, hence, apply the equilibrium relation obtained in the quasi-magnetostatic case, viz. $M=\frac{\chi_{M}}{1+\chi_{M}}\cdot \frac{1}{\mu_{0}}B.$ Then, we have to solve

(117a) $$\begin{array}{ccccc} \mathrm{div}(B) & =0\;, & -\left(1+\chi \right)\varepsilon_{0}\partial_{t}\left(\nabla \varphi \right)+n^{F}\upsilon +J^{F} & =\frac{1}{\mu_{0}}\mathrm{curl}\left(\frac{1}{1+\chi_{M}}B\right)\;, & \end{array}$$

(117b) $$\begin{array}{cccc} \left[\;[B\cdot \nu ]\;\right] & =0\;, & -\nu \times \left[\;\left[\underset{s}{\upsilon }\;\;\times \left(\left(1+\chi \right)\varepsilon_{0}\nabla \varphi \right)\right]\;\right]+\underset{s}{J}\;{\;}^{F} & =\frac{1}{\mu_{0}}\nu \times \left[\;\left[\frac{1}{1+\chi_{M}}B\right]\;\right]\;, \end{array}$$

and can evaluate the electric current as

(118) $$\begin{array}{cc} j^{e} & =n^{F}\upsilon +J^{F}-\partial_{t}\left(\chi \varepsilon_{0}\nabla \varphi \right)+\frac{1}{\mu_{0}}\mathrm{curl}\left(\frac{\chi_{M}}{1+\chi_{M}}B\right)\;. \end{array}$$

For a paramagnetic and a diamagnetic material, typically, $|\chi_{M}|\ll 1$, and thus, the Lorentz magnetisation is small, such that it often might be appropriate to neglect the magnetic term, making the evaluation of the electric current $j^{e}$ available already without the solution of (117).

## Figures and Tables

**Table 1:** Scaling of variables and constitutive functions in the bulk regions and on the surface.

x	$=x^{\mathrm{ref}}\;\cdot \overset{˘}{x}$ ,	*t*	$=t^{\mathrm{ref}}\;\cdot \overset{˘}{t}$ ,	υ	$=\frac{x^{\mathrm{ref}}}{t^{\mathrm{ref}}}\;\cdot \overset{˘}{\upsilon }$ ,
E	$=E^{\mathrm{ref}}\cdot \overset{˘}{E}$ ,	E	$=E^{\mathrm{ref}}\cdot \overset{˘}{E}$ ,	P	$=\varepsilon_{0}E^{\mathrm{ref}}\cdot \overset{˘}{P}$ ,
B	$=B^{\mathrm{ref}}\cdot \overset{˘}{B}$ ,	M	$=\frac{1}{\mu_{0}}B^{\mathrm{ref}}\cdot \overset{˘}{M}$ ,	M	$=\frac{1}{\mu_{0}}B^{\mathrm{ref}}\cdot \overset{˘}{M}$ ,
$n^{F}$	$=e_{0}n^{\mathrm{ref}}\;\cdot {\overset{˘}{n}}^{F}$ ,	$J^{F}$	$=e_{0}n^{\mathrm{ref}}\frac{x^{\mathrm{ref}}}{t^{\mathrm{ref}}}\;\cdot {\overset{˘}{J}}^{F}$ ,	$J^{e}$	$=e_{0}n^{\mathrm{ref}}\frac{x^{\mathrm{ref}}}{t^{\mathrm{ref}}}\;\cdot {\overset{˘}{J}}^{e}$ ,
$\sigma^{\mathrm{EM}}$	$=k_{B}T^{\mathrm{ref}}\;n^{\mathrm{ref}}\;\cdot \overset{˘}{\sigma^{\mathrm{EM}}}$ ,	k	$=k_{B}T^{\mathrm{ref}}\;n^{\mathrm{ref}}\frac{1}{x^{\mathrm{ref}}}\;\cdot \overset{˘}{k}$ ,	π	$=k_{B}T^{\mathrm{ref}}\;n^{\mathrm{ref}}\frac{1}{t^{\mathrm{ref}}}\;\cdot \overset{˘}{\pi }$ ,
$\underset{s}{n}\;{\;}^{F}$	$=e_{0}\underset{s}{n}\;{\;}^{\mathrm{ref}}\;\cdot \overset{˘}{n}{}^{F}$ ,	$\underset{s}{J}\;{\;}^{F}$	$=e_{0}\underset{s}{n}\;{\;}^{\mathrm{ref}}\frac{x^{\mathrm{ref}}}{t^{\mathrm{ref}}}\;\cdot \overset{˘}{\underset{s}{J}\;\;}{}^{F}$ ,	$\underset{s}{J}\;{\;}^{e}$	$=e_{0}\underset{s}{n}\;{\;}^{\mathrm{ref}}\frac{x^{\mathrm{ref}}}{t^{\mathrm{ref}}}\;\cdot \overset{˘}{\underset{s}{J}\;\;}{}^{e}$ ,
		$\underset{s}{k}\;\;$	$=k_{B}T^{\mathrm{ref}}\;\underset{s}{n}\;{\;}^{\mathrm{ref}}\frac{1}{x^{\mathrm{ref}}}\;\cdot \overset{˘}{k}$ ,	$\underset{s}{\pi }\;\;$	$=k_{B}T^{\mathrm{ref}}\;\underset{s}{n}\;{\;}^{\mathrm{ref}}\frac{1}{t^{\mathrm{ref}}}\;\cdot \overset{˘}{\pi }$ ,

## Appendix A. Balance of Inner Energy

Multiplication of the total mass balance by $-\frac{1}{2}{\left|\upsilon \right|}^{2}$, scalar multiplication of the momentum balance by υ, and subsequent addition yield the balance of kinetic energy:

(A1a) $$\begin{array}{ccc} \partial_{t}(\frac{1}{2}{\rho |\upsilon |}^{2})+\mathrm{div}\left(\frac{1}{2}\rho {\left|\upsilon \right|}^{2}\upsilon -\left(\Sigma -\sigma^{\mathrm{EM}}\right)\upsilon \right)= & -(\Sigma -\sigma^{\mathrm{EM}}):\nabla \upsilon +\rho f\cdot \upsilon +k\cdot \upsilon \;, & \\ \partial_{t,\nu }\left(\frac{1}{2}\underset{s}{\rho }\;\;{\left|\underset{s}{\upsilon }\;\;\right|}^{2}\right)+{\left(\frac{1}{2}\underset{s}{\rho }\;\;|\underset{s}{\upsilon }\;\;{|}^{2})\underset{s}{\upsilon }\;{\;}_{\tau }^{\Delta }-\underset{s}{\sigma }\;{\;}^{i\Delta }\underset{s}{\upsilon }\;{\;}^{i}\right)}_{∥\Delta }\; & \end{array}$$

(A1b) $$\begin{array}{cc} -2k_{M}\underset{s}{\upsilon }\;{\;}_{\nu }\;\left(\frac{1}{2}\underset{s}{\rho }\;\;{\left|\underset{s}{\upsilon }\;\;\right|}^{2}\right)= & -\underset{s}{\sigma }\;{\;}^{i\Delta }\underset{s}{\upsilon }\;{\;}_{∥\Delta }^{i}+\underset{s}{\rho }\;\;\underset{s}{f}\;\;\cdot \underset{s}{\upsilon }\;\;+\underset{s}{k}\;\;\cdot \underset{s}{\upsilon }\;\;\\ \; & -[\;[\frac{1}{2}{\rho |\upsilon |}^{2}\left(\upsilon_{\nu }-\underset{s}{\upsilon }\;{\;}_{\nu }\right)-\left(\Sigma -\sigma^{\mathrm{EM}}\right)\upsilon \cdot \nu ]\;]\\ \; & +[\;[\frac{1}{2}\rho |\upsilon -\underset{s}{\upsilon }{\;\;|}^{2}\left(\upsilon_{\nu }-\underset{s}{\upsilon }\;{\;}_{\nu }\right)-\left(\Sigma -\sigma^{\mathrm{EM}}\right)\left(\upsilon -\underset{s}{\upsilon }\;\;\right)\cdot \nu ]\;]\;. \end{array}$$

Adding the kinetic energy density and the inner energy density, we obtain the energy density of matter as $\rho e=\rho u+\frac{1}{2}\rho {\left|\upsilon \right|}^{2}$. Then, the postulation of the total energy of the field and matter being a conserved quantity in the absence of gravitation implies the forcing terms in the balance of ρe as the Joule heat according to (13). We split the heat flux as $Q-q^{\mathrm{EM}}$, where the second term vanishes in the absence of an electromagnetic field.

(A2a) $$\begin{array}{cc} \partial_{t}\left(\rho e\right)+\mathrm{div}\left(\rho e\;\upsilon +\left(Q-q^{\mathrm{EM}}\right)-\left(\Sigma -\sigma^{\mathrm{EM}}\right)\upsilon \right)= & \;\pi +\rho f\cdot \upsilon \end{array}$$

(A2b) $$\begin{array}{cc} \partial_{t,\nu }\left(\underset{s}{\rho }\;\;\underset{s}{e}\;\;\right)+(\left(\underset{s}{\rho }\;\;\underset{s}{e}\;\;\right)\underset{s}{\upsilon }\;{\;}_{\tau }^{\Delta }+\underset{s}{q}\;{\;}^{\Delta }-\underset{s}{\sigma }\;{\;}^{i\Delta }\underset{s}{\upsilon }\;{\;}_{i}{)}_{∥\Delta }-2k_{M}\underset{s}{\upsilon }\;{\;}_{\nu }\;\left(\underset{s}{\rho }\;\;\underset{s}{e}\;\;\right)= & \;\underset{s}{\pi }\;\;+\underset{s}{\rho }\;\;\underset{s}{f}\;\;\cdot \underset{s}{\upsilon }\;\;\\ -[\;[\rho e\left(\upsilon_{\nu }-\underset{s}{\upsilon }\;{\;}_{\nu }\right)\; & +\left(\left(Q-q^{\mathrm{EM}}\right)-\left(\Sigma -\sigma^{\mathrm{EM}}\right)\upsilon \right)\cdot \nu ]\;] \end{array}$$

Subtracting (A1) from (A2) and setting $q^{\mathrm{EM}}=E\times M$ then yield the inner energy balance (51). The choice of $q^{\mathrm{EM}}$ is motivated by the fact that it makes the production terms in the inner energy balance directly computable within either of the Galilean limits of the Maxwell equations, as demonstrated below, without having to rely on the additional remaining Maxwell equations.

### Appendix A.1. Quasi-Electrostatic Limit

In the quasi-electrostatic limit, we have E=E, and it holds

(A3) $$\begin{array}{cccc} \pi -k\cdot \upsilon & =J^{F}\cdot E+J^{P}\cdot E\;, & \underset{s}{\pi }\;\;-\underset{s}{k}\;\;\cdot \underset{s}{\upsilon } & =\underset{s}{J}\;{\;}^{F}\cdot \bar{E}+\underset{s}{J}\;{\;}^{P}\cdot \bar{E}\;. \end{array}$$

In the volume, we use that, in this limit, curl(E)=0 and apply

(A4a) $$\begin{array}{ccc} J^{P}\cdot E\; & =\left(\partial_{t}P+\upsilon \mathrm{div}\left(P\right)+\mathrm{curl}\left(M\right)+\mathrm{curl}\left(P\times \upsilon \right)\right)\cdot E\;, & \end{array}$$

(A4b) $$\begin{array}{ccc} \mathrm{div}(E\times M)\; & =-\mathrm{curl}(M)\cdot E\;, & \end{array}$$

(A4c) $$\begin{array}{cc} \mathrm{curl}(P\times \upsilon )\; & =\mathrm{div}(\upsilon )P+(\upsilon \cdot \nabla )P-\mathrm{div}(P)\upsilon -(P\cdot \nabla )\upsilon \;, \end{array}$$

to obtain (52a) from

(A5) $$\begin{array}{cc} \pi -k\cdot \upsilon +\mathrm{div}(E\times M)\; & =J^{F}\cdot E+\left(\partial_{t}P+\left(\upsilon \cdot \nabla \right)P\right)\cdot E+\mathrm{div}\left(\upsilon \right)\;P\cdot E-\left(P\cdot \nabla \right)\upsilon \cdot E\;. \end{array}$$

On the surface, we use that ν×[[E]]=0 and that (34) implies

(A6) $$\begin{array}{cc} \underset{s}{J}\;{\;}^{P}\cdot \bar{E}\; & =\nu \times \left[\;\left[M\cdot E\right]\;\right]-\nu \times [\;\left[\left(\left(\upsilon -\underset{s}{\upsilon }\;\;\right)\times P\right)\cdot E\right]\;]\;, \end{array}$$

to obtain (52b) from

(A7) $$\begin{array}{cc} \underset{s}{\pi }\;\;-\underset{s}{k}\;\;\cdot \underset{s}{\upsilon }\;\;+\left[\;\left[E\times M\right]\;\right]\cdot \nu \; & =\underset{s}{J}\;{\;}^{F}\cdot \bar{E}-\left[\;\left[P\cdot \nu \;\left(\upsilon -\underset{s}{\upsilon }\;\;\right)\cdot E-\left(\upsilon -\underset{s}{\upsilon }\;\;\right)\cdot \nu \;P\cdot E\right]\;\right]\;. \end{array}$$

### Appendix A.2. Quasi-Magnetostatic Limit

In the quasi-magnetostatic limit, we have M=M, and it holds

(A8) $$\begin{array}{ccccc} \pi -k\cdot \upsilon & =\left(J^{F}+J^{P}\right)\cdot \left(\upsilon \times B\right)\;, & \; & \underset{s}{\pi }\;\;-\underset{s}{k}\;\;\cdot \underset{s}{\upsilon } & =\left(\underset{s}{J}\;{\;}^{F}+\underset{s}{J}\;{\;}^{P}\right)\cdot \left(\underset{s}{\upsilon }\;\;\times \bar{B}\right)\;. \end{array}$$

In the volume, we use that $\mathrm{curl}\left(E\right)=-\partial_{t}B$ is not small and apply

(A9a) $$\begin{array}{ccc} J^{P}\cdot \left(\upsilon \times B\right)\; & =\mathrm{curl}(M)\cdot (\upsilon \times B)\;, & \end{array}$$

(A9b) $$\begin{array}{ccc} \mathrm{div}(E\times M)\; & =-\left(\partial_{t}B+\mathrm{curl}\left(B\times \upsilon \right)\right)\cdot M-\mathrm{curl}\left(M\right)\cdot \left(\upsilon \times B\right)\;, & \end{array}$$

(A9c) $$\begin{array}{cc} \mathrm{curl}(B\times \upsilon )\; & =\mathrm{div}(\upsilon )B+(\upsilon \cdot \nabla )B-(B\cdot \nabla )\upsilon \;, \end{array}$$

to obtain (53a) from

(A10) $$\begin{array}{cc} \pi -k\cdot \upsilon +\mathrm{div}(E\times M)\; & =J^{F}\cdot \left(\upsilon \times B\right)-\left(\partial_{t}B+\left(\upsilon \cdot \nabla \right)B\right)\cdot M-\mathrm{div}\left(\upsilon \right)\;B\cdot M+\left(B\cdot \nabla \right)\upsilon \cdot M\;. \end{array}$$

On the surface, we use $\nu \times [\;\left[\underset{s}{\upsilon }\;\;\times B\right]\;]=0$ and

(A11) $$\begin{array}{cc} \underset{s}{J}\;{\;}^{P}\cdot \left(\underset{s}{\upsilon }\;\;\times \bar{B}\right)\; & =\nu \times \left[\;\left[M\right]\;\right]\cdot \left(\underset{s}{\upsilon }\;\;\times \bar{B}\right)=\nu \times \left[\;\left[M\cdot \left(\underset{s}{\upsilon }\;\;\times B\right)\right]\;\right]\\ \; & =\nu \times \left[\;\left[M\cdot \left(\upsilon \times B\right)\right]\;\right]-\nu \times [\;\left[M\cdot \left(\left(\upsilon -\underset{s}{\upsilon }\;\;\right)\times B\right)\right]\;]\\ \; & =\nu \times \left[\;\left[M\cdot E\right]\;\right]-\nu \times [\;\left[\left(\left(\upsilon -\underset{s}{\upsilon }\;\;\right)\times B\right)\cdot M\right]\;] \end{array}$$

to obtain (53b) from

(A12) $$\begin{array}{cc} \underset{s}{\pi }\;\;-\underset{s}{k}\;\;\cdot \underset{s}{\upsilon }\;\;+\left[\;\left[E\times M\right]\;\right]\cdot \nu \; & =\underset{s}{J}\;{\;}^{F}\cdot \left(\underset{s}{\upsilon }\;\;\times \bar{B}\right)-\left[\;\left[B\cdot \nu \;\left(\upsilon -\underset{s}{\upsilon }\;\;\right)\cdot M-\left(\upsilon -\underset{s}{\upsilon }\;\;\right)\cdot \nu \;B\cdot M\right]\;\right]\;. \end{array}$$
